# Serum Abnormal Metabolites for Evaluating Therapeutic Response and Prognosis of Patients With Multiple Myeloma

**DOI:** 10.3389/fonc.2022.808290

**Published:** 2022-02-28

**Authors:** Yujun Wei, Jinying Wang, Fei Chen, Xin Li, Jiajia Zhang, Man Shen, Ran Tang, Zhongxia Huang

**Affiliations:** ^1^ Multiple Myeloma Medical Center of Beijing, Department of Hematology, Beijing Chao-yang Hospital, Capital Medical University, Beijing, China; ^2^ Multiple Myeloma Medical Center of Beijing, Beijing Chao-yang Hospital, Capital Medical University, Beijing, China

**Keywords:** multiple myeloma, UPLC-MS, metabolome, biomarkers, treatment, disease progression, prognosis

## Abstract

**Aims:**

To evaluate abnormal metabolites related to treatment response and prognosis of multiple myeloma (MM) patients through ultra performance liquid chromatography tandem mass spectrometry (UPLC-MS).

**Methods:**

Forty-six symptomatic MM patients were included in this study who had a prior high level of positive monoclonal proteins before receiving targeted therapy with bortezomib-based regimens. UPLC-MS along with traditional immunofixation was performed on MM diagnostic samples and effective serum samples, and UPLC-MS was used to target valuable metabolic markers related to M protein.MM patients were segregated into pre-therapy (pre-T) and post-therapy (post-T) groups according to the response after chemotherapy. A monoclonal protein could be detected at baseline in 33 newly diagnosed MM (NDMM), 13 refractory and relapsed MM (RRMM) patients and 20 healthy controls (HC) by immunofixation.

**Results:**

Between pre-T and post-T patients, the data showed that 32, 28 and 3 different metabolites were significantly correlated with M protein in IgG, IgA and light chain-type MM, respectively. These identified metabolites were significantly enriched in arginine and proline metabolism as well as glycerophospholipid metabolism pathways. Among them, PC (19:0/22:2) was displayed to increase significantly and consistently with M protein in each subtype of MM after treatment, which obviously indicated that it was related to the treatment response of MM. Further survival analysis of metabolic markers found that aspartic acid, LysoPE (16:0), SM (d18:1/17:0), PC (18:0/24:1), PC (16:0/16:0), TG (18:1/18:1/22:5) and LysoPE (18:2) reaching a certain cutoff value may be associated with shorter progression free survival (PFS). Finally, Cox multivariate regression analysis identified three factors were independent prognostic factors of MM. Moreover, there were significantly different in PC (19:0/22:2) and in aspartic acid between MM patients and healthy people.

**Conclusion:**

This work identified significant metabolic disorders in 46 pairs off pre- and post-therapy MM patients, specifically in arginine, proline and glycerophospholipid pathways. The abnormal metabolites have the potential to serve as new biomarkers for evaluating treatment response and prognosis, as well as early monitoring of disease activity. Therefore, these systematic studies on abnormal metabolites as biomarkers for diagnosis and treatment will provide the evidence for future precise treatment of MM.

## Introduction

Multiple myeloma (MM) is characterized by abnormal proliferation of clonal plasma cells in bone marrow that secrete monoclonal immunoglobulin or its fragment (M protein), causing damage to corresponding organs and tissues (1). The incidence of MM has been increasing in recent years (2), and the median age of diagnosis is 69 years (3, 4). Prior to diagnosis, MM patients generally have two stages of monoclonal gammopathy of undetermined significance (MGUS) and asymptomatic myeloma (SMM) (5). According to International Myeloma Working Group (IMWG), the diagnosis for MM requires bone marrow clonal plasma cells≥10% and one symptom of CRAB (hypercalcemia, renal dysfunction, anemia, or osteolytic bone damage) (3, 6). In recent years, with the clinical application of proteasome inhibitors (PIs), immunomodulators (IMiDs), and other new tumor targeted drugs, the average survival time of MM patients has been extended to 5–7 years (7, 8). The use of multi-color flow cytometry (MFC) or second-generation sequencing (NGS) technology to detect minimal residual disease (MRD), along with other technologies, further enhances the selection of treatment options as well as the assessment of disease status for diagnosis and recurrence (9). Although treatment and survival have been improved, MM remains incurable.

MM is a disease that is highly dependent on the tumor microenvironment (TME) (10, 11). In MM, adipocytes in bone marrow account for 70% of the total cell volume; free fatty acids (FFA) secreted by adipocytes enable myeloma cells to obtain more energy from aerobic oxidation, which promotes tumor growth (11, 12). Tumor cells promote tumorigenesis by reprogramming energy metabolism (12).

CRAB symptoms and M protein determination are important indices for clinically evaluating MM activity, and are also important indicators of treatment response. When MM patients have extensive extramedullary disease (EMD), clonal plasma cells may be characterized by plasmablasts and lack of M protein secretion. Given this, the tumor load and therapeutic response should not be judged by M protein. Conventional imaging examination also lacks quantitative indicators. Therefore, it would be of great value to develop a noninvasive and convenient detection method for MM, to be used clinically as a supplement for evaluating disease recurrence or progression and to predict the efficacy and prognosis of chemotherapeutic treatments.

Serum metabology is a powerful tool for exploring biomarkers and drug targets. Previous studies by our research team found that the serum metabolites of MM patients are significantly different from those of healthy people with 12 metabolites. Therefore, it shows that this type of serum metabolite with significant changes can be used as a new marker for evaluating MM (13). A study by Gonsalves et al. (14), which utilized an untargeted metabolite and targeted complex lipid profiling of bone marrow(BM) plasma identified that the metabolites of branch chain amino acids (BCAAs), 3-hydroxy-kynurenine, phosphatidylethanolamines (PE), lactosylceramides (LCER) and phosphatidylinositols (PI), were significantly different across each group between patients with MGUS and MM.

Changes in serum metabolism characteristics can be detected in solid tumors before and after treatment using liquid chromatography with tandem mass spectrometry (LC/MS-MS) to effectively predict the therapeutic effect of chemotherapy drugs (15). However, few studies have analyzed whether abnormal metabolites can be used to evaluate therapeutic response in MM. In this study, ultra performance liquid chromatography tandem mass spectrometry (UPLC-MS/MS) was used to study changes in abnormal metabolites pre and post therapy in patients with symptomatic MM. The goal of this study was to determine the relationship between abnormal metabolites, treatment response, and prognosis for MM patients. The study also aimed to further expand the quantitative index for judging MM disease progression and treatment response, beyond the standard use of M protein.

## Materials and Methods

### Study Subjects

MM patients were diagnosed at Beijing Chao-yang Hospital (Western campus) at Capital Medical University from August 2018 to December 2020. Diagnosis and response criteria were based on the IMWG diagnostic criteria (3). Symptomatic MM patients received initial therapy with a bortezomib-based regimen for one to eight cycles (average four to six cycles). In patients achieving a complete response (CR) or partial response (PR), the regimen was repeated for two to four cycles or autologous stem cell transplantation was completed as consolidation therapy. In the absence of any response, the treatment regimen was modified. Maintenance therapy, when utilized, consisted of lenalidomide at a dose of 25 mg administered orally, every other day.

Forty-six pairs of serum samples were collected from MM patients pre-therapy(pre-T) and post-therapy(post-T), and were analyzed by metabonomics. The cases included 33 newly diagnosed multiple myeloma patients (NDMM) and 13 refractory recurrent multiple myeloma patients (RRMM). The general clinical features of the patients were shown in **Tables 1** and **2**.

**Table 1:** Baseline clinical characteristics of 33 NDMM patients pre-and post-therapy.

Items	Groups		P Value
	Pre-T	Post-T	
Numbers	33	33	/
Age(years)	61.3	61.3	/
Gender: M/F(cases)	22/11	22/11	/
**Type of MM**			
Ig G/Ig A/k/λ/NS(cases)	5/11/12/4/1	5/11/12/4/1	/
Stage of R-ISS: I/II/III(cases)	4/12/17	4/12/17	/
MM with EMD(cases,%)	10(39.3%)	10(39.3%)	/
**High-risk cytogenetics in FISH**			
No. of high-risk genes 0/1/2	5/4/14	5/4/14	/
1q21+/t(4;14)/t(14;16)/del(17p) (cases)	11/6/7/8	11/6/7/8	/
BMPC (%)	46.33 (74.75)*	0.50 (1.25)*	P=0.043
Calcium (mmol/L,C)	2.26 (0.23)*	2.20 (0.16)*	P=0.035
Creatinine (μmol/L,R)	76.00 (110.5)*	77.00 (44.5)*	P=0.017
Hemoglobin (g/dL,A)	9.60 ± 2.23	11.46 ± 2.11	P<0.001
Bone lesion (B) (cases,%)	28(84.85%)	28(84.85%)	/
LDH(U/L)	176.6 (67)*	195.3 (59.6)*	P=0.156
β 2-MG (mg/L)	5.03 (8.47)*	3.68 (2.36)*	P<0.001
Albumin (g/L)	36.30 (9.00)*	38.00 (5.50)*	P=0.130
Globulin(G/L)	26.00 (26.80)*	23.30 (6.90)*	P<0.001
Triglyceride (mmol/L)	1.28 (0.84)*	1.50 (1.14)*	P=0.124
Uric acid (μmol/L)	418.59 ± 147.13	329.97 ± 110.96	P=0.008
Cholesterol (mmol/L)	3.89 ± 0.94	4.94 ± 1.48	P=0.004
HDL-C (mmol/L)	1.07 ± 0.27	1.27 ± 0.27	P=0.001
LDL-C (mmol/L)	2.20 ± 0.75	2.85 ± 1.08	P=0.012

M, male; F, female; MM, multiple myeloma; NDMM, newly diagnosed MM; pre-T, pre-therapy group; post-T,post-therapy group; Ig G, immunoglobulin G; Ig A, immunoglobulin A; k, k light chain; λ, λ light chain;NS, non-secretory; BMPC, plasma cell in bone marrow; LDH, Lactatedehydrogenase;β 2-MG, β 2-microglobulin; RRMM, relapsed patients with MM; R-ISS, Revised International Staging System; FISH, fluorescence in situ hybridization; LDH, Lactate dehydrogenase; TC, total cholesterol; TG, triglyceride; HDL-C, high-density lipoprotein cholesterol; LDL-C, low-density lipoprotein cholesterol; Fish,fluorescence in situ hybridization; 0,no above genetic abnormalities; 1,one genetic abnormality; 2, more than two genetic abnormalities.

*The data with non-normal distribution is represented by the median (interquartile range q3-q1), which cannot be written in the format of mean ± standard deviation.

**Table 2:** Baseline clinical characteristics of 13 RRMM patients pre -and post-therapy.

Items	Groups		P Value
	Pre-T	Post-T	
Numbers	13	13	/
Age(years)	64.90 ± 10.50	64.90 ± 10.50	/
Gender: M/F(cases)	7/6	7/6	/
**Type of MM**			
IgG/IgA/k/λ/NS(cases)	8/2/2/0/1	8/2/2/0/1	/
Stage of R-ISS: I/II/III(cases)	0/3/10	0/3/10	/
MM with EMD(cases)	6(60%)	6(60%)	/
**High-risk cytogenetics in FISH**			
No. of high-risk genes 0/1/2	1/2/2	1/2/2	/
1q21+/t(4;14)/t(14;16)/del(17p) (cases)	4/2/2/0	4/2/2/0	/
BMPC (%)	38 (59.50)*	3.75 (1.25)*	P=0.019
Calcium (mmol/L,C)	2.34 ± 0.23	2.20 ± 0.23	P=0.122
Creatinine (μmol/L,R)	71.45 (34.9)*	67.6 (24.33)*	P=0.099
Hemoglobin (g/dL,A)	9.98 ± 2.91	11.66 ± 2.54	P=0.013
Bone lesion (B) (cases,%)	13(100%)*	13(100%)*	/
LDH(U/L)	181.36 ± 55.64	174.63 ± 36.59	P=0.637
β 2-MG (mg/L)	4.08 (3.22)*	3.44 (2.25)*	P=0.034
Albumin (g/L)	31.39 ± 4.85	33.99 ± 6.95	P=0.184
Globulin(G/L)	53.94 ± 29.15	29.59 ± 11.11	P=0.012
Triglyceride(mmol)	1.62 ± 0.71	1.83 ± 1.09	P=0.386
Uric acid (μmol/L)	389.09 ± 110.11	349.54 ± 91.42	P=0.391
Cholesterol(mmol/L)	3.98 ± 1.46	4.65 ± 0.97	P=0.101
HDL-C (mmol/L)	1.05 ± 0.27	1.17 ± 0.20	P=0.236
LDL-C (mmol/L)	2.29 ± 1.01	2.69 ± 0.83	P=0.077

M, male; F, female; MM, multiple myeloma; RRMM, relapsed and refractory MM; pre-T, pre-therapy group; post-T,post-therapy group; Ig G, immunoglobulin G; Ig A, immunoglobulin A; k, k light chain; λ, λ light chain; NS, non-secretory; BMPC, plasma cell percent in bone marrow; LDH, Lactatedehydrogenase;β 2-MG, β 2-microglobulin; R-ISS, Revised International Staging System; FISH, fluorescence in situ hybridization; LDH, Lactate dehydrogenase; TC, total cholesterol; TG, triglyceride; HDL-C, high-density lipoprotein cholesterol; LDL-C, low-density lipoprotein cholesterol; Fish, fluorescence in situ hybridization; 0,no above genetic abnormalities; 1,one genetic abnormality; 2, more than two genetic abnormalities.

*The data with non-normal distribution is represented by the median (interquartile range q3-q1), which cannot be written in the format of mean ± standard deviation.

### Groups

**Figure 1:**
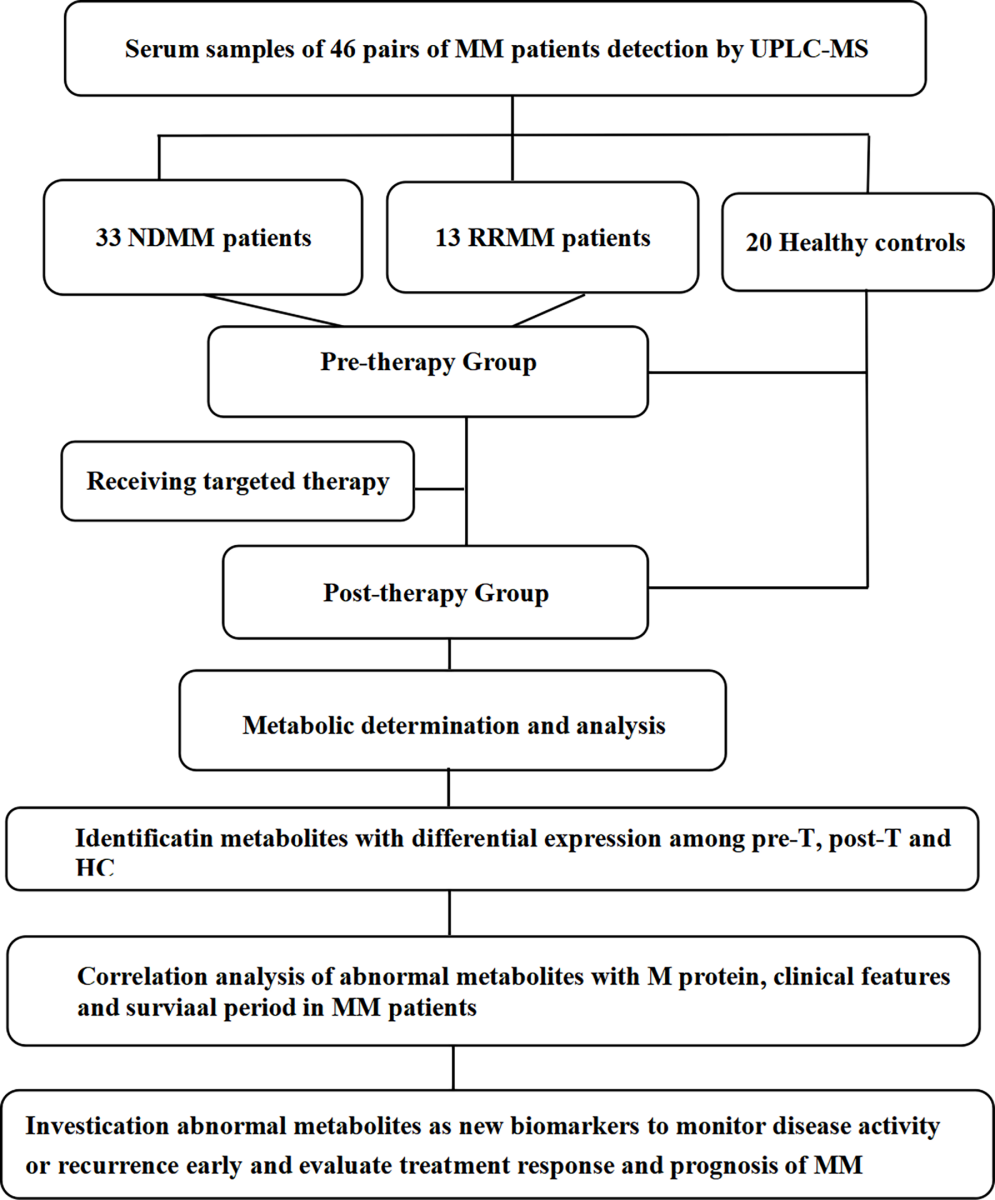
The workflow of our work. MM, multiple myeloma; pre-T, pre-therapy; post-T, post-therapy; HC, healthy controls; UPLC-MS, ultra performance liquid chromatography tandem mass spectrometry.

MM patients were divided into a pre-therapy(pre-T) group and a post-therapy(post-T) group; post-therapy(post-T) group refers to patients who achieved at least PR after treatment (3). Additionally, 20 healthy gender- and age-matched subjects were selected as healthy controls (HCs). The specific sample situation from the patients were shown in **Figure 1**.

The pre-therapy(pre-T) group was defined by the following standards: 1) symptomatic myeloma with no induction chemotherapy in the past three months; and 2) presence of active CRAB symptoms, including newly diagnosed MM patients and recurrent MM patients. Bone marrow puncture or M protein identification were completed within 1 week of admission, and 4 ml venous blood was collected at that time.

Post-therapy(post-T) group included symptomatic myeloma patients who required treatment after admission and who received 2–4 courses of bortezomib-based chemotherapy (on average). In most of the post-T patients, treatment resulted in improved crab symptoms and a decrease in M protein to a level indicative of PR, at which point venous blood was collected for the study.

The following cases were excluded from this study: 1) cases with less than two courses of chemotherapy; 2) cases in which M protein decreased below PR levels; 3) patients with plasma cell leukemia or amyloidosis; and 4) patients with a history of hyperlipidemia.

Preparation of serum samples as well as determination and analysis of metabolism by UPLC-MS has been previously described (13).

### Pathway Enrichment Analysis

MetaboAnalyst 4.0 (http://www.metaboanalyst.ca/faces/ModuleView.xhtml) was used for pathway enrichment analysis to identify the biological role of metabolites in MM.

### Data Processing and Statistical Analysis

To identify metabolites with differential expression, the original data were analyzed by skyline quantitative analysis software from The University of Washington. First, unsupervised principal component analysis (PCA) was used to analyze standard (QC) samples along with other samples for quality control evaluation. Next, the sample analysis was divided into PCA and orthogonal partial least squares discriminant analysis (OPLS-DA).

In this experiment, SPSS 22.0 and GraphPad 8.0 software were used for statistical analysis and drawing. Considering that some data did not conform to normality, Spearman correlation analysis was applied. Differential expression of metabolites among pre-T, post-T, and HC was analyzed by GraphPad in a violin plot using the Wilcoxon test (for two groups of data not conforming to normal), a paired sample t-test, or two independent sample t-tests. Sensitivity and specificity of metabolic markers were analyzed by receiver operating characteristic (ROC) curve. The Kaplan-Meier survival analysis model was used to establish the relationship between metabolic markers and disease progression. Overall survival (OS) was the time from diagnosis to death, and progression free survival (PFS) was the time from chemotherapy to tumor progression or death. The Cox proportional hazards regression model was used to analyze the factors influencing disease progression.

### Ethics

This work was approved by the Ethics Committee of Beijing Chao-yang Hospital, Capital Medical University, and informed written consent was obtained from all patients and healthy individuals. The research complied with the principles of the Declaration of Helsinki.

## Results

For the NDMM patient column, the average age was 61.3 years, and the male to female ratio was 2:1(22:11),as shown in **Table 1**. We found that the blood lipid level of MM patients decreased before chemotherapy and recovered after treatment. For example, compared to post-T, the NDMM patient’s cholesterol (3.89 ± 0.94 vs 4.94 ± 1.48, P = 0.004), high-density lipoprotein (1.07 ± 0.27 vs 1.27 ± 0.27, P = 0.001), and low-density lipoprotein (2.20 ± 0.75 vs.2.85 ± 1.08, P = 0.012) increased, therefore there was a statistically difference in baseline clinical data before and after treatment in NDMM. Similarly, in **Table 2** for RRMM patients column, there were differences in plasma cell % in bone marrow (BMPC),hemoglobin, globulin, LDL-C, and β 2-MG between the pre-T and the post-T group (**Table 2**).

### Analysis of the Difference and Reliability of Abnormal Metabolites Between Groups

**Figure 2:**
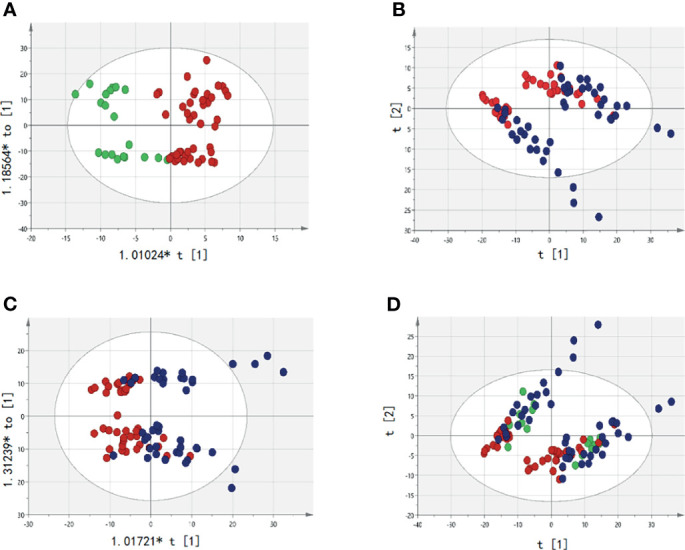
Analysis of serum abnormal metabolites by PCA and OPLS-DA. The red dot represents the active group, namely Pre-T(pre-therapy group);The blue dots represent the relatively stable disease group after treatment, namely Post-T(post-therapy group);The green dots represent healthy controls (HC). **(A)** It represents the difference between pre-T and HC; **(B)** It represents the difference between pre-T and post-T in PCA; **(C)** It represents the difference between pre-T and post-T (OPLS-DA); **(D)** It represents the metabolites were significantly changed pre-T, post-T, and HC in PCA, which indicates that patients in pre-T has a tendency to change to HC after treatment.

The samples detected by UPLC-MS were divided into PCA and OPLS-DA. The markers were then filtered and confirmed by combining the results of the VIP values (VIP > 1) and t-test (P < 0.05), fc > 1.15, fc < 0.85. There were metabolic differences among HC, pre-T, and post-T, as shown in **Figure 2**. Differences in abnormal metabolites between MM patients and HCs suggested the occurrence of MM, or the recurrence and progression of disease.

Between pre-T and post-T groups, there were 219 differentially expressed metabolites in the paired serum samples of 46 MM patients. Among the 33 NDMM patients, there were 20 differentially expressed metabolites. As for the 13 RRMM patients column, there were 50 differentially expressed metabolites, including triglyceride, lysophosphatidylcholine, lecithin, and sphingomyelin. In addition, differences were identified for Cer (d18:1/22:1), hexosylceramide (Hex2Cer) (d18:2/24:0), and undecylic acid (**Figure 2** and **Figure S1**). In order to better present metabolite differences before and after treatment, Venn diagrams were plotted among NDMM, RRMM, and all MM patients. This suggested that four common metabolites were screened out, including PC (16:0/16:0), LysoPE (16:0), LysoPE (18:1), and PC (19:0/22:2) (**Figure S2**). Given this, the abnormal metabolites appeared to be related to treatment response of MM, suggesting that metabolite analysis is a useful tool for management of MM.

In comparing pre-T patients with HC, some metabolic pathways were significantly enriched in MM, including the arginine biosynthesis pathway, the β-alanine metabolism pathway, the histidine, D-glutamine and D-glutamate metabolism pathway. In comparing pre-T patients with post-T, pre-therapy patients of NDMM were significantly enriched in the arginine biosynthesis pathway, histidine metabolism pathway, glycerol phospholipid metabolism pathway, arginine, and proline biosynthesis pathway. These findings were similar for RRMM patients before therapy, who showed significant enrichment in the glycerol phospholipid metabolic pathway, as shown in **Figure S3**.

Compared with HC, a total of 162 metabolites were found to be differentially expressed in pre-T patients. These potential biomarkers were then filtered and confirmed by combining the results of the VIP values (VIP > 1) and t-test (P < 0.05), fc > 1.15, fc < 0.85. We further analyzed the top nine metabolites from the ROC curve, and evaluated the diagnostic value of each metabolite, as shown in **Figure S4**, including aspartic acid (AUC = 0.8840, P = 0.0005), glutamic acid (AUC = 0.9040, P = 0.0002), montanic acid (AUC = 0.8080, P = 0.0049), Hex2 cerd (18:2/24:0); (AUC = 0.8920, P = 0.0003), PE (O-18:1/18:2) (AUC = 0.8440,P = 0.0017), PC (17:0/18:0) (AUC = 0.8320, P = 0.0024), PC (19:0/22:2) (AUC = 0.9800, P < 0.0001), PC (20:2/20:0) (AUC = 0.9360, P < 0.0001), and SM (d-18:2/24:0) (AUC = 0.9840, P < 0.0001). However, the diagnostic values of PC (16:0/16:0), LysoPE(16:0), LysoPE (18:1) had no statistical significance. The diagnostic reliability of PC (19:0/22:2) and SM (d-18:2/24:0) reached 98%, followed by PC (20:2/20:0), glutamic acid, Hex2 cerd (18:2/24:0), and aspartic acid, with a diagnostic reliability of approximately 90%. This suggested that screening for these six substances had the potential to aid in the diagnosis of MM.

### Abnormal Metabolites for Evaluating Disease Activity and Treatment Response in MM Subtypes

**Figure 3:**
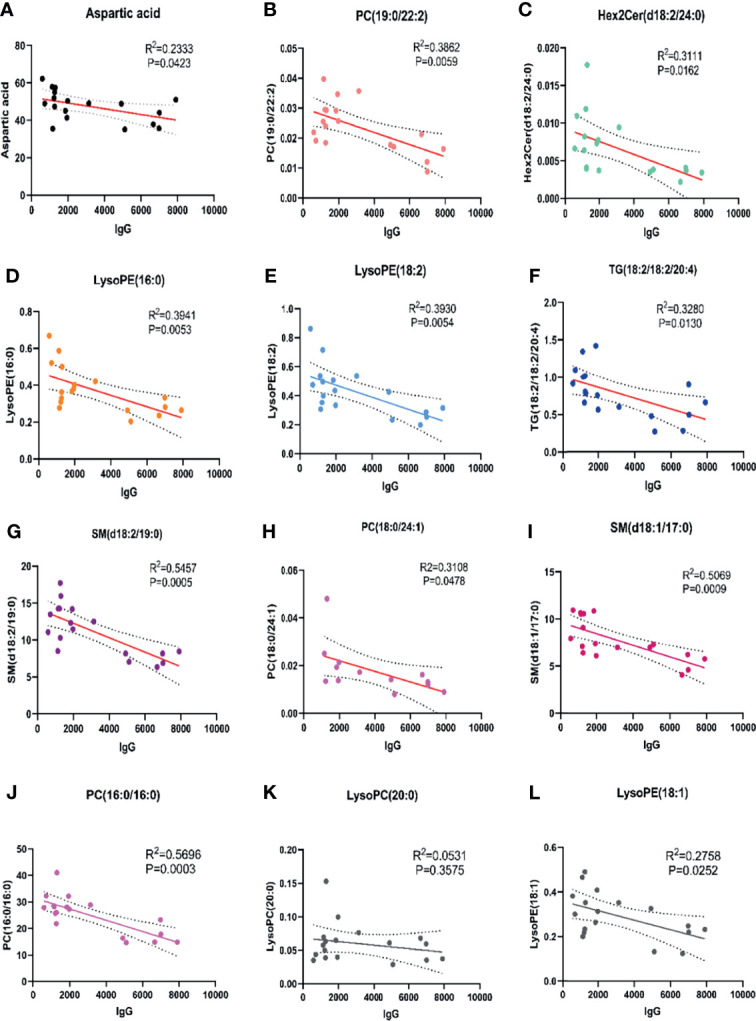
Correlation analysis of abnormal metabolites with M protein in IgG type MM. There were 9 abnormal metabolites related to M protein in patients with IgG type MM. **(A)** Aspartic acid (R^2^ = 0.2333, P=0.0423); **(B)** PC (19:0/22:2) (R^2^ = 0.3862, P=0.0059); **(C)** Hex2 cerd (18:2/24:0) (R^2^ = 0.3111, P=0.0162); **(D)** LysoPE (16:0) (R^2^ = 0.3941, P=0.0053); **(E)** LysoPE (18:2) (R^2^ = 0.3930, P=0.0054); **(F)** TG (18:1/18:1/20:4) (R^2^ = 0.3280, P=0.0130); **(G)** SM (d18:2/19:0) (R^2^ = 0.5457, P=0.0005); **(H)** PC (18:0/24:1) (R^2^ = 0.3108, P=0.0478); **(I)** SM (d18:1/17:0) (R^2^ = 0.5069, P=0.0009); **(J)** PC (16:0/16:0) (R^2^ = 0.5696, P=0.0003); **(K)** LysoPC (20:0) (R^2^ = 0.0531, P=0.3575); **(L)** LysoPE (18:1) (R^2^ = 0.2758, P=0.0252).

**Figure 4:**
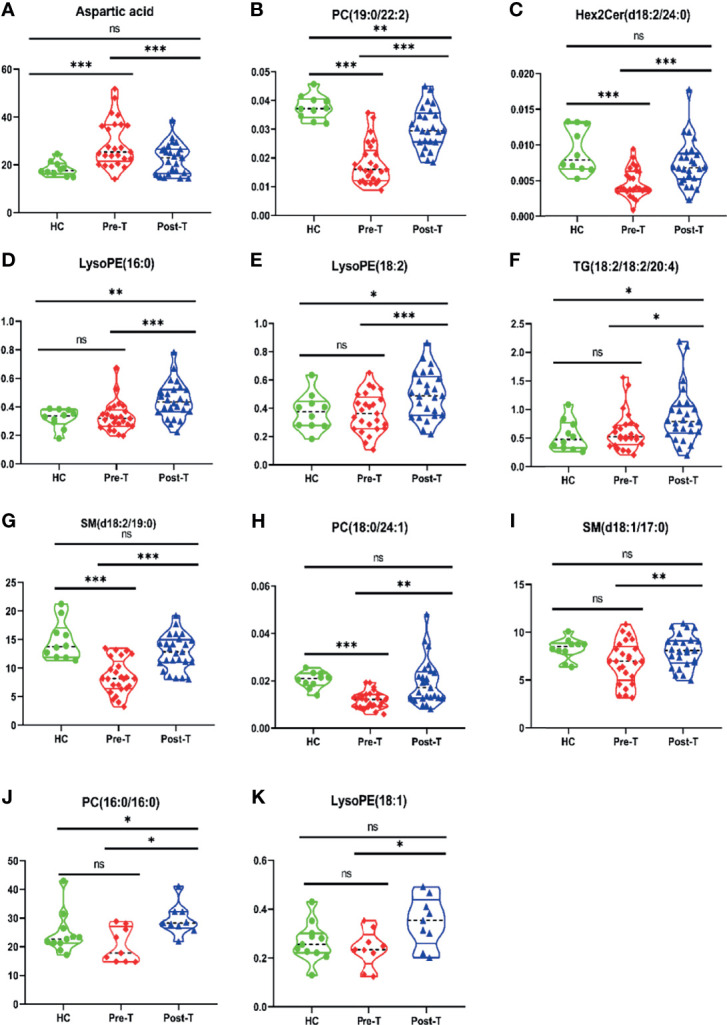
Differences of metabolites related to M protein in IgG-type MM patients through Violin Plot. There were differences of 9 metabolites related to M protein in Pre-T (pre-therapy group) and Post-T (post-therapy group) in IgG type MM patients, compared with HC. **(A)** Aspartic acid; **(B)** PC (19:0/22:2); **(C)** Hex2 cerd (18:2/24:0); **(D)** LysoPE (16:0); **(E)** LysoPE (18:2); **(F)** TG (18:1/18:1/20:4); **(G)** SM (d18:2/19:0); **(H)** PC (18:0/24:1); **(I)** SM (d18:1/17:0); **(J)** PC (16:0/16:0); **(K)** LysoPE (18:1). *P < 0.05, **P < 0.01, ***P < 0.001, ns P > 0.05.

Among 32 metabolites significantly related to M protein (P<0.01) in IgG-type MM, we identified 12 substances that were closely negatively correlated with M protein: aspartic acid (R2 = 0.2333, P = 0.0423), PC (19:0/22:2) (R2 = 0.3862, P = 0.0059), Hex2 cerd (18:2/24:0) (R2 = 0.3111, P = 0.0162), LysoPE (16:0) (R2 = 0.3941, P = 0.0053), LysoPE (18:2) (R2 = 0.3930, P = 0.0054), TG (18:1/18:1/20:4) (R2 = 0.3280, P = 0.0130), SM (d18:2/19:0) (R2 = 0.5457, P = 0.0005), PC 18:0/24:1 (R2 = 0.3108, P = 0.0478), and SM (d18:1/17:0) (R2 = 0.5069, P = 0.0009), PC (16:0/16:0) (R^2^ = 0.5696, P=0.0003), LysoPC (20:0) (R^2^ = 0.0531, P=0.3575), LysoPE (18:1) (R^2 =^ 0.2758, P=0.0252), as shown in **Figure 3**. Aspartic acid was increased in pre-T patients compared to HC and the following metabolites were decreased: PC (19:0/22:2), Hex2 Cerd (18:2/24:0), SM (d18:2/19:0), and PC (18:0/24:1), suggesting their important diagnostic value in MM. The LysoPE (16:0), LysoPE (18:2), TG (18:1/18:1/20:4), SM (d18:1/17:0), PC (16:0/16:0), and LysoPE (18:1) showed no changes between multiple myeloma and healthy patients. Between post-T and HC, there were differences in PC (19:0/22:2), LysoPE (16:0), LysoPE (18:2), PC (16:0/16:0), and TG (18:1/18:1/20:4) (**Figure 4**). More importantly, there were obvious differences in the above 11 metabolites between pre-T and post-T patients, representing the treatment response. The following five metabolites may have value not only for diagnosis but also for evaluating the treatment response in IgG-type MM: aspartic acid, PC (19:0/22:2), Hex2 Cerd (18:2/24:0), SM (d18:2/19:0), and PC (18:0/24:1). In particular, PC (19:0/22:2) was not only able to identify patients with NDMM, but was also able to distinguish pre-T with improved patients after therapy, which has important clinical significance. It was also found that PC (16:0/16:0) was consistent with our previous studies (13).

In IgA-type MM, our data showed that 28 metabolites in pre-T and post-T were significantly correlated with M protein (P < 0.01). The data were displayed after comprehensive analysis of six substances with the highest correlation, and which negatively correlated with M protein: PC (O-22:0/22:4; R2 = 0.5297, P = 0.0073), PC (16:0/20:5; R2 = 0.4158, P = 0.0236), PC (18:0/24:1; R2 = 0.3845, P = 0.0315), PC (O-16:1/20:4; R2 = 0.5376, P = 0.0067), PC (19:0/22:2; R2 = 0.3597, P = 0.0393), and SM (d18:3/22:2, R2 = 0.4371, P = 0.0192), respectively, as shown in **Figure S5**. All differences were presented in a violin plot, as shown in **Figure S6**. For pre-T patients compared with HC, five metabolites were decreased, including PC (O-22:0/22:4; P < 0.00), PC (18:0/24:1; P < 0.001), PC (O-16:1/20:4; P < 0.001), PC (19:0/22:2; P < 0.001), and SM (d18:3/22:2; P < 0.01). These data highlight the value for the diagnosis of IgA-type MM using these metabolites. More importantly, the levels increased after treatment, indicating the value of these metabolites for evaluating treatment response in MM patients. Similarly, it was found that PC (O-16:1/20:4), PC (O-22:0/22:4), PC (19:0/22:2), and SM (d18:3/22:2) may be valuable not only for diagnosis but also for evaluating the treatment response in IgA-type MM.

In patients with light chain-type MM, inductive analysis identified three substances that were negatively correlated with the ratio of free light chain: TG 18:1/18:1/22:5 (R2 = 0.3184, P = 0.0183), PE 18:1/18:2 (R2 = 0.2455, P = 0.0431), and PC 19:0/22:2 (R2 = 0.2961, P = 0.0196) (**Figure S7**). All differences are displayed in **Figure S8**. As with the above two types of MM, it was found that there was dual value in diagnosis and evaluation of treatment response for PC (19:0/22:2).

Upon comparing differential expression of metabolites across disease stages, TG (18:1/18:1/22:5) and TG (18:2/18:2/20:4) were found to be significantly lower in stage III than in stage II (P < 0.05). These data demonstrate the variable expression of metabolites in different MM disease stages (**Figure S9**).

With the exception of one case of non-secretory MM, the other nine case of NDMM combined with extensive extramedullary disease (EMD) patients were analyzed. Three screened metabolic markers, including LysoPE (16:0), TG (18:1/18:1/22:5), and Aspartic acid, showed no significant differences between patients with or without EMD. This may suggest that there was no metabolite difference between patients with EMD and other MM patients. However, the comparison before and after treatment showed that there were differences in the above three metabolites. This suggests that patients with EMD showed the same metabolite differences as other MM patients while received treatment with bortezomib- based regimens (**Figure S10**).

M protein is the general standard for evaluating disease activity and treatment response in MM. Therefore, the relationship between these metabolites and efficacy was based on M protein as the standard in the current study, and the two showed consistent changes before and after treatment, as in **Figure S11**.

### Abnormal Metabolites for Evaluating Prognosis of MM Patients

**Figure 5:**
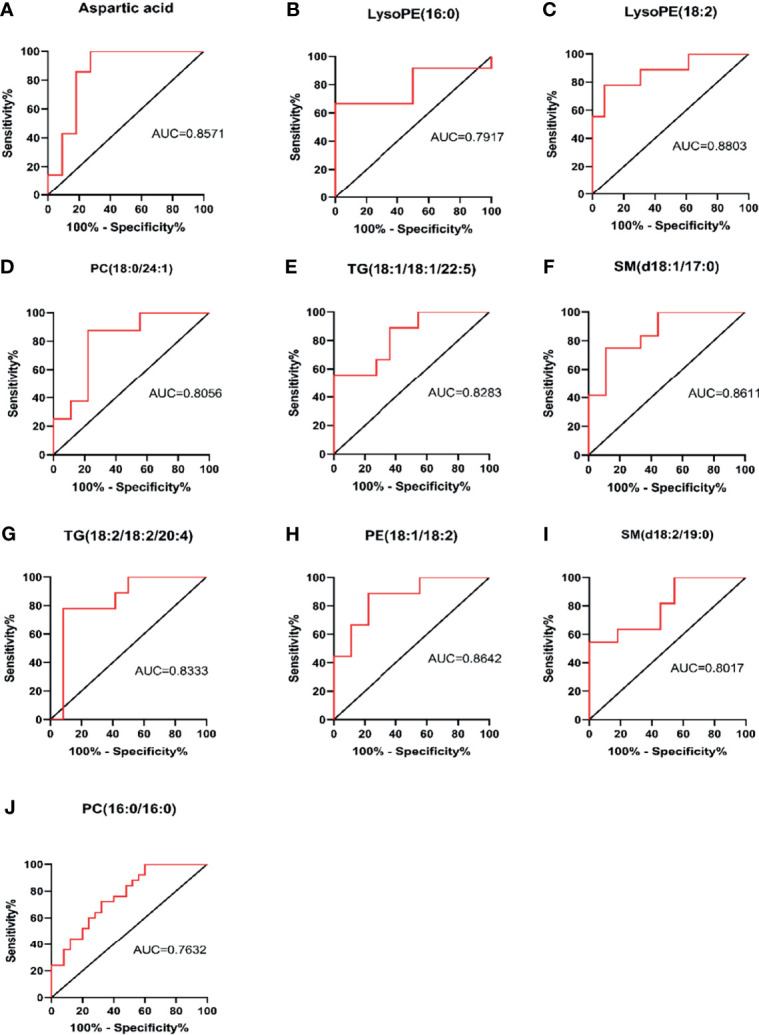
Sensitivity and specificity analysis of metabolic markers for assessing disease progression by ROC curve. PC, phosphatidylcholine (lecithin); PE, phosphatidylethanolamine (cephalin); TG, triglyceride; SM, sphingomyelin; LysoPE, hemolytic cephalin. **(A)** Aspartic acid; **(B)** LysoPE (16:0); **(C)** LysoPE (18:2); **(D)** PC (18:0/24:1); **(E)** TG (18:1/18:1/22:5)); **(F)** SM (d18:1/17:0); **(G)** TG (18:2/18:2/20:4; **(H)** PE (18:1/18:2); **(I)** SM, (d18:2/19:0); **(J)** PC (16:0/16:0).

The area under the curve (AUC value) is typically used to reflect the reliability of evaluating disease progression. The closer the AUC value is to 1, the higher the accuracy of diagnosing a disease. The ROC curves of some metabolic markers were as follows: aspartic acid (AUC = 0.8571), LysoPE (16:0) (AUC = 0.7917), LysoPE (18:2) (AUC = 0.883), PC (18:0/24:1) (AUC = 0.8056), TG (18:1/18:1/22:5) (AUC = 0.8283), SM (d18:1/17:0) (AUC = 0.8611), TG (18:2/18:2/20:4) (AUC = 0.8333), PE (18:1/18:2) (AUC = 0.8642), SM (d18:1/19:0) (AUC = 0.8017), and PC(16:0/16:0) (AUC = 0.7632,**Figure 5**). ROC curve analysis showed that these 10 metabolic markers had high sensitivity and specificity in evaluating disease progression.

**Figure 6:**
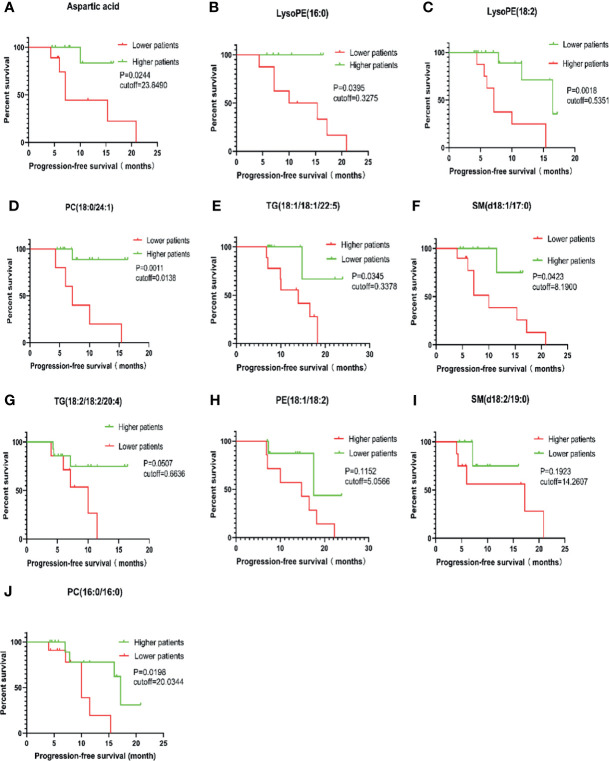
Survival analysis of PFS in 46 cases of MM patients. PC, phosphatidylcholine (lecithin); PE, phosphatidylethanolamine (cephalin); TG, triglyceride; SM, sphingomyelin; LysoPE, hemolytic cephalin. **(A)**, Aspartic acid; **(B)**, LysoPE (16:0); **(C)**, LysoPE (18:2); **(D)**, PC (18:0/24:1); **(E)**, TG (18:1/18:1/22:5)); **(F)**, SM (d18:1/17:0); **(G)**, TG (18:2/18:2/20:4; **(H)**, PE (18:1/18:2); **(I)**, SM (d18:2/19:0); **(J)**, PC (16:0/16:0).

In order to further evaluate the prognostic factors, Kaplan-Meier survival analysis was performed for M protein-related metabolites. Considering the short follow-up time, PFS was selected as the end event of the study. Upon completing survival analysis, we found that aspartic acid, LysoPE (16:0), LysoPE(18:2), PC (18:0/24:1), TG (18:1/18:1/22:5), SM (d18:1/17:0), and PC(16:0/16:0) were related to the progression of MM or were useful as metabolic markers to assist in clinical evaluation of the prognosis of MM (**Figure 6**).

The results also showed that patients with aspartate levels less than 23.8490 were more likely to develop disease than those with levels above the cutoff value (P = 0.0244). Similar results were found for LysoPE (16:0) < 0.3275, SM (d18:1/17:0) < 8.1900, PC (18:0/24:1) < 0.0138, and PC (16:0/16:0) < 20.0344. Lower levels for these metabolites indicated a higher likelihood for developing disease or relapsing. However, the opposite was true regarding LysoPE (18:2) > 0.5351 and TG (18:1/18:1/22:5) > 0.3378; higher values for these metabolites favored MM disease progression or recurrence.

### Independent Prognostic Factor Analysis

Univariate Cox regression analysis was performed first and all factors that could affect the prognosis of MM were included, The P value was < 0.2 for the following seven variables: hemoglobin, albumin, RISS stage, lysoPE (16:0), PE (18:1/18:2), TG (18:1/18:1/22:5), and Aspartic acid. The variables were reconfirmed, and multivariate Cox regression analysis was next carried out.This ultimately concluded that three factors were potentially related to early progression and adverse prognosis of MM, such as RISS stage (P = 0.044, 95%CI: 1.059-97.528), LysoPE (16:0) (P = 0.012, 95%CI: 1.141-517.139) and TG (18:1/18:1/22:5) (P = 0.025, 95%CI: 1.473-320.438).However, the 95%CI was wide, which may be related to shorter follow-up (**Table S1**).

### Analysis of Relation of BMPC With Abnormal Metabolites

Between pre-T and post-T patients, the data showed that different metabolites were significantly correlated with M protein in MM subtypes such as in IgG, IgA and light chain-type MM. Subsequently, relationship between the percent of plasma cells (PC) in BM (BMPC%) with the metabolites was analyzed, and showed that BMPC% were positively correlated with aspartic acid (R^2^ = 0.2730, P = 0.0151), PC 16:0/16:0 (R^2^ = 0.2732, P = 0.0181) and PC (O-22:0/22:4; R^2^ = 0.4389, P = 0.0015), and no correlation with other metabolites, as in **Figure S12**.

## Discussion

In this study, we found that cholesterol and the levels other lipids were decreased before treatment in patients with symptomatic MM. We also found that the abnormal lipid levels returned to normal range when the disease reached partial response after treatment. Pei et al. (16) previously reported that the fat content of bone marrow decreased by MRI, and a large sample retrospective study from the Korean reported consistent findings (17).

Further ROC curve analysis identified significant differences in the reliability of PC (19:0/22:2) (phosphatidylcholine, lecithin) and SM (d-18:1/24:0) (sphingomyelin) in the diagnosis of MM, which was as high as 98%. ROC analysis also identified differences in PC (20:2/20:0), glutamic acid, Hex2 cerd (18:2/24:0), and aspartic acid. The diagnostic reliability of MM was close to 90%, suggesting that screening for the above six substances could aid in the diagnosis of MM. Compared with HC, levels of glutamic acid and aspartic acid were increased, while the levels of PC (19:0/22:2), SM (d-18:1/24:0), PC (20:2/20:0) and Hex2 cerd (18:2/24:0) were decreased in MM. Previous studies involving lung cancer and colorectal cancer all support the diagnostic value of serum abnormal metabolites for tumor invasion (18–21). Our previous studies and some MS studies of MM have also found significant differences between NDMM, RRMM, and healthy individuals. Taken together, this reveals diagnostic value for abnormal serum metabolites in understanding MM activity and suggests that these metabolites have the potential to be used as biomarkers for MM diagnosis (13, 18).

In the present study, PC (O-16:1/20:4), PC (O-22:0/22:4), PC (19:0/22:2), and SM (d18:3/22:2) showed significant differences in IgA MM before and after therapy. For IgG type MM column, aspartic acid, PC (19:0/22:2), Hex2 cerd (18:2/24:0), LysoPE (16:0), LysoPE (18:2), and SM (d18:2/19:0); TG (18:1/18:1/22:5) and PC (19:0/22:2) were the same as in for light chain MM. These changes were consistent with that of M protein, suggesting that they can be used to judge the therapeutic response or curative effect of MM. Additionally, PC (19:0/22:2) was shown to be associated with M protein in IgA, IgG, and light chain-type MM. The metabolite was also increased significantly after treatment, indicating that PC (19:0/22:2) was of great significance in evaluating therapeutic response. Furthermore, Leonor et al. (22) reported that changes in metabonomics before and after treatment may be helpful for objectively monitoring the response to treatment in 27 patients with NDMM, which is consistent with our data.

Extensive extramedullary disease (EMD) may occur at the initial diagnosis or in the process of disease progression of MM, which is an aggressive subentity of poor prognosis of MM. So far, the pathogenesis of EMD is unknown. In addition to TME abnormalities such as homing disorders, a review by Bhutani et al. (23) suggested that the development of EMD may be associated with tumor subclones independent of TME. However, our present study did not show that metabolic abnormalities were the unique pathological mechanism of EMD. At the beginning, patients with EMD may respond to the initial treatment of bortezomib and other new drugs, but they may not avoid the development of escape of apoptosis and therapeutic resistance. Furthermore, they tend to acquire a more aggressive disease in later stages (24, 25), which is consistent with the clinical treatment response observed in the real world.

In the present study, we found that levels of aspartic acid, LysoPE (16:0), LysoPE (18:2), PC (18:0/24:1), TG (18:1/18:1/22:5), and SM (d18:1/17:0) were associated with shorter PFS (median PFS, 9.57 months), which may be related to early progression or recurrence of MM. Yang et al. (19) previously proposed that changes in metabolites before and after treatment were helpful for judging the prognosis of lung cancer.

Our study also demonstrated that lipid levels of MM patients were lower than that of healthy people. After treatment, cholesterol, high-density lipoprotein, and low-density lipoprotein were increased to varying degrees in MM patients. Moreover, patients with TG (18:1/18:1/22:5) > 0.3378 showed shorter PFS time than those with TG (18:1/18:1/22:5) lower than the cutoff value (P = 0.0345).

Multivariate Cox regression analysis showed that TG (18:1/18:1/22:5) was an independent prognostic factor for MM. Anti-obesity drugs such as etoposide and orlistat inhibit fatty acid synthesis and plasma cell proliferation, thereby leading to a reduction in the viability of human myeloma cells. As such, targeting fatty acid metabolism may represent a new therapeutic direction for MM (22).

Here, Hex2 cerd (18:2/24:0) showed significant differences before and after treatment in patients with IgG-type MM, and also showed a linear correlation with M protein. These findings suggest that hexosylceramide can be used as a new marker to judge the relapse of RRMM or a discriminant indicator of MM treatment response.

Differences were also found for lysophosphatidylethanolamine and triglyceride (TG). LysoPE (16:0) and TG (18:1/18:1/22:5) were negatively correlated with M protein, and significant differences were observed in pre-T and post-T. ROC curve analysis revealed the diagnostic value of this metabolite for MM and survival analysis confirmed that it was correlated with prognosis. Furthermore, patients with LysoPE (16:0) < 0.3275 were more likely to experience disease progression, and patients with TG (18:1/18:1/22:5) > 0.3378 had shorter PFS. Cox regression analysis showed that LysoPE (16:0) (P = 0.009) and TG (18:1/18:1/22:5) (P = 0.018) were associated with early disease progression and poor prognosis. Expression of hexcer (hexosylceramide) has been shown to be significantly higher in cholangiocarcinoma tissues than in paracancerous tissues (20) and is also associated with shorter survival times.

The above abnormal hexosylceramide and sphingomyelin metabolism is related to the “sphingomyelin ceramide cycle,” which may be involved in the pathological microenvironment of energy metabolism and immune metabolism in MM. By affecting nerve conduction, this could result in poorer survival and prognosis in MM.

Compared with the HC, metabolic pathways were significantly enriched for arginine, β-Alanine, alanine, aspartate, glutamate, D-glutamine, and D-glutamate, and these changes are consistent with the post-therapy patients. Furthermore, aspartic acid was found to be linearly correlated with M protein and to be increased in NDMM patients, contrary to non-progressing patients. Next, ROC curve analysis further confirmed the value of aspartic acid for evaluating the early progression of MM. Finally, patients with aspartic acid value < 23.8490 were found to be more prone to disease progression than those with higher aspartic acid values. Therefore, aspartic acid has the potential be used as a biomarker for the diagnosis and evaluation of treatment response in patients with myeloma. We provided verification research from lung cancer (19, 26); additionally, Li et al. (27) found that a high level of branched chain amino acids promoted the progression of pancreatic ductal adenocarcinoma, suggested that metabolic abnormalities may be involved in tumorigenesis. In tumor metabolism, cancer cells have a stronger ability to obtain energy through glucose and fat, followed by T lymphocytes, and reprogramming energy. In contrast, cancer cells have the highest glutamine uptake and amino acid metabolism to support cell growth (28). The expression of asparagine synthetase has been associated with poor prognosis (29). Meanwhile, fatty acid synthesis affected the metabolic reprogramming of activated T cells mediated by mTOR, which is an important part of the differentiation of CD4 + effector T (Teff)cells. Regulatory T (Treg) cells primarily depend on fatty acids from β- oxidation during development (30). The levels of glutamic acid and aspartic acid were significantly increased in bone marrow plasma of MM patients, and glutamine was significantly decreased. Further RNA sequencing showed a higher relative expression of c-myc and glutamine transporters (such as ASCT2 and SN2) in MM tumor cells compared to MGUS (31). Taken together, an abnormal TME with energy metabolism, immune disorders, and genetic instability leads to tumorigenesis and poor prognosis of MM.

Although myeloma cells have the characteristics of focal distribution, and M protein in blood or urine is routinely used as an indicator of tumor load and treatment response of MM, it should be more objective to comprehensively evaluate the therapeutic effect if the above two are combined.

The current study analyses the relation of BMPC with abnormal metabolites in the MM subtype. It appears that more myeloma cells in BM were associated with high serum levels of abnormal metabolites such as aspartic acid, PC 16:0/16:0 and PC(O-22:0/22:4). However, in the complex TME, from MGUS to symptomatic MM, the proportion of myeloma cells increases and other cells such as bone marrow stromal cells and immune cells gradually develop in a direction that is more conducive to tumor growth. Therefore, the producing cell of abnormal metabolites requires further evaluation by more in-depth studies. With the accumulation of abnormal metabolites in TME, it will inhibit T cell function (32), lead to the reprogramming of multiple metabolic pathways in MM (33), which is conducive to the formation and progression of tumors (34).

This is a rare systematic study from a single center. Forty-six pairs of MM patients before and after treatment provided evidence for better consistency between abnormal metabolites and M protein, which enabled identification of new biomarkers to monitor disease activity or early recurrence, and evaluate treatment response and prognosis of MM. Inevitably, there are limitations to the present study. First, the difference of metabolite profiling among different periods of disease evolution could not be compared due to the small sample size. Second, the correlated relation of BMPC% with three abnormal metabolites was analyzed, However, the metabolite-producing cells were not explored. Third, the regulatory mechanism of metabolite production or the relationship between metabolism and immune cells reprogramming could not be further revealed. In our future work, the metabolite profiling will compare MM patients in different stages or subtype including MGUS, NDMM and RRMM. Furthermore, the metabolite biomarkers for treatment response or drug resistance will be identified and the potential abnormal metabolite-producing cells, the regulatory mechanism of immune cell reprogramming will be further validated through a large clinical cohort or deep basic science and clinical study.

## Conclusion

In short, we identified significant metabolic differences before and after treatment in 46 paired serum samples from MM patients and the healthy. Additional analysis demonstrated that these metabolic markers have important value and should be recommended for evaluation of early disease progression, treatment response and prognosis. It is reasonable to assume that the complex disorder of tumor metabolism and immune microenvironments may be conducive to the occurrence and progression of MM. Thus, inhibition of abnormal metabolites presents a novel potential approach for future precision treatment of MM. Therefore, efforts are underway to better understand the detailed relation between metabolism and immune in MM, and further basic research studies are required to provide experimental evidence.

## Data Availability Statement

The original contributions presented in the study are included in the article/**Supplementary Material**, further inquiries can be directed to the corresponding author.

## Ethics Statement

The studies involving human participants were reviewed and approved by Medical Ethics Committee, Beijing Chao-yang Hospital, Capital Medical University. The patients/participants provided their written informed consent to participate in this study.

## Author Contributions

The project was designed and experiments were conducted by ZH. The data were collected primarily by YW and JW, assisted by FC, XL, JZ, MS, and RT. Statistical analysis and manuscript writing were mainly completed by YW and ZH. All authors agreed to publish the manuscript in this journal.

## Funding

The work was supported by Science and Technology Project of Beijing Science and Technology. Commission (Z171100000417010), and the construction project on key medical disciplines of Shijingshan District, Beijing.

## Conflict of Interest

The authors declare that the research was conducted in the absence of any commercial or financial relationships that could be construed as a potential conflict of interest.

## Publisher’s Note

All claims expressed in this article are solely those of the authors and do not necessarily represent those of their affiliated organizations, or those of the publisher, the editors and the reviewers. Any product that may be evaluated in this article, or claim that may be made by its manufacturer, is not guaranteed or endorsed by the publisher.
