# Supplementary material for: Serum Abnormal Metabolites for Evaluating Therapeutic Response and Prognosis of Patients With Multiple Myeloma
Source: Front Oncol. 2022 Feb 28;12:808290. doi: 10.3389/fonc.2022.808290 (PMC8919723; doi:10.3389/fonc.2022.808290)
Supplement: Supplementary file 1 [file DataSheet_1.zip › Data Sheet 1/Illustrations of supplementary tables and figures 20220118.docx]

**Illustrations of supplementary tables and figures**

**TABLE S1. Independent prognostic factors analysis in 46 MM patients by multivariate Cox Regression.** Univariate Cox Regression analysis was performed first and all factors that could affect the prognosis of MM were included, The P value was < 0.2 for the following seven variables: hemoglobin, albumin, RISS stage, lysoPE (16:0), PE (18:1 / 18:2), TG (18:1 / 18:1 / 22:5), and Aspartic acid. The variables were reconfirmed, and multivariate Cox regression analysis was next carried out for independent prognostic factors analysis. This ultimately concluded that three factors were potentially related to early progression and adverse prognosis of MM, such as RISS stage (P = 0.044, 95%CI: 1.059-97.528), LysoPE (16:0) (P = 0.012, 95%CI: 1.141-517.139) and TG (18:1/18:1/22:5) (P = 0 .025, 95%CI: 1.473-320.438).

**Note**: Cr,creatinine; LysoPE,lysophosphatidylcholine; PE,phosphatidylethanolamine (cephalin);TG, triglyceride;PC,phosphatidylcholine (lecithin);Arginine, aspartic acid.* According to the RISS staging criteria of the International Myeloma Working Group

**FIGURE S1. Metabolites analysis pre- and post-therapy of RRMM patients in Heat map**.The abscissa stand for the comparison of samples pre-T and post-T in RRMM patients, each small cell represents a sample, and the ordinate represents the measured metabolites.The darker the red color is, the higher the abundance is, and the darker the blue color is, the lower the abundance is.

**Note:** C,phosphatidylcholine(lecithin);PE,phosphatidylethanolamine(cephalin);TG,triglyceride; SM,sphingomyelin;LysoPC,lysophosphatidylcholine;LysoPE,lysophosphatidylcholine; Cer (d18:1 / 22:1) ,ceramide; hex2cer (d18:2 / 24:0) , hexosylceramide.

**FIGURE S2. Analysis of metabolite differences pre- and post-therapy by Venn diagrams.** There were 219, 20 and 50 metabolites of difference between pre-T and post-T(DPPT) across all MM ,NDMM and RRMM patients. Among them, there were four metabolites that overlapped in three circles, and four that overlapped in two circles between NDMM and RRMM.

**Note**: DPPT, differences of metabolites between pre-T and post-T; **A**, DPPT of NDMM and RRMM patients; **B**, DPPT of NDMM, RRMM patients and all MM patients.

**FIGURE S3. Enrichment maps for pathways of metabolites.**

**Note**: **(A)** Compared with HC, the arginine biosynthesis pathway, β-alanine metabolism pathway, histidine metabolism pathway, D-glutamine, and D-glutamate were significantly enriched of MM patients pre-therapy; **(B)** Compared with post-T, the arginine biosynthesis pathway, histidine metabolism pathway, glycerol phospholipid metabolism pathway, arginine and proline biosynthesis pathway were significantly enriched of NDMM patients pre-therapy; **(C)** Compared with post-T, the glycerol phospholipid metabolic pathway were significantly enriched of RRMM patients pre-therapy.

**FIGURE S4. Analyze the diagnostic reliability of metabolic markers by ROC curves.** In Comparing abnormal metabolites of pre-T with HC, the metabolites significantly changed in the pre-therapy group (pre-T) of total MM patients.

Note: **(A)**, aspartic acid (AUC=0.8840, P=0.0005); **(B)**, Glutamic acid（AUC=0.9040，P=0.0002）; **(C)**, Montanic acid（AUC=0.8080，P=0.0049）; **(D)**, Hex2 cerd(18:2/24:0) （AUC=0.8920，P=0.0003）; **(E)**, PE(O-18:1/18:2)（AUC=0.8440，P=0.0017）; **(F)**, PC(17:0/18:0)（AUC=0.8320，P=0.0024）; **(G)**, PC(19:0/22:2) （AUC=0.9800，P＜0.0001）; **(H)**, PC(20:2/20:0) （AUC=0.9360，P＜0.0001）; **(I)**, SM(d-18:2/24:0) （AUC=0.9840，P＜0.0001）.

**FIGURE S5.**  **Correlation analysis of scatter plot between metabolites and M protein in IgA type MM**. Six metabolites associated with M protein were found in IgA type MM patients.

**Note**: **(A)**, PC(O-22:0/22:4)（R^2^=0.5297，P=0.0073）; **(B)**, PC(16:0/20:5) （R^2^=0.4158，P=0.0236）; **(C)**, PC（18:0/24:1）（R^2^=0.3845，P=0.0315）; **(D)**, PC(O-16:1/20:4) （R^2^=0.5376，P=0.0067）; **(E)**, PC(19:0/22:2) （R^2^=0.3597，P=0.0393）; **(F)**, SM（d18:3/22:2）（R^2^=0.4371，P=0.0192）.

**FIGURE S6.** **Differences of metabolites related to M protein in IgA type MM patients through Violin Plot**. There were differences of six metabolites related to M protein in Pre-T and Post-Tin IgA type MM patients, compared with HC.

**Note: (A)**, PC(O-22:0/22:4); **(B)**, PC(16:0/20:5); **(C)**, PC（18:0/24:1）; **(D)**, PC(O-16:1/20:4); **(E)**, PC(19:0/22:2); **(F)**, SM(d18:3/22:1). * P < 0.05, * * P < 0.01, * * * P < 0.001, ns P > 0.05.

**FIGURE S7.**  **Correlation analysis between metabolites and the ratio of sFLC in scatter plot.** In patients with light chain-type MM, inductive analysis identified three substances that were negatively correlated with the ratio of sFLC: TG 18:1/18:1/22:5 (R2 = 0.3184, P = 0.0183), PE 18:1/18:2 (R2 = 0.2455, P = 0.0431), and PC 19:0/22:2 (R2 = 0.2961, P = 0.0196).

**Note**: **(A)**, TG(18:1/18:1/22:5); **(B)**, PE(18:1/18:2); **(C)**, PC(19:0/22:2); **sFLC, seurm free light chain.**

**FIGURE S8.**  **Differences of metabolites related to the ratio of sFLC in light chain-type MM patients through Violin Plot**.

**Note: (A),** TG(18:1/18:1/22:5); **(B),** PE(18:1/18:2); **(C),** PC(19:0/22:2). * P < 0.05, * * P < 0.01, * * * P < 0.001, ns P > 0.05.

**FIGURE S9. Relationship between abnormal metabolites and RISS stages in 46 MM patients.** Upon comparing differential expression of metabolites across disease stages of R-ISS, TG (18:1/18:1/22:5) and TG (18:2/18:2/20:4) were found to be significantly lower in stage III than in stage II (P < 0.05), as in **Figure S9**.

**Note：**A, Aspartic acid，B, Hex2 cerd(18:2/24:0)，C,LysoPE(16:0);D,LysoPE(18:2);E,TG（18:1/18:1/22:5）; F,TG（18:2/18:2/20:4）.* P < 0.05, , ns P > 0.05.

**Figure S10.** **Analysis of metabolites in NDMM patients combined with EMD patients.** With the exception of one case of non-secretory MM, the other nine case of NDMM with extensive extramedullary disease (EMD) patients were analyzed. Three screened metabolic markers, including LysoPE(16:0), TG(18:1/18:1/22:5), and Aspartic acid, showed no significant differences between patients with or without EMD. However, the comparison before and after treatment showed that there were differences in the above three metabolites.

**Note:** EMD**,** extensive extramedullary diseases; **(A),** LysoPE (16:0); **(B),** TG(18:1/18:1/22:5) ; **(C),** Aspartic acid. * P < 0.05, * * P < 0.01, * * * P < 0.001, ns P > 0.05.

**Figure S11.** **Consistency of metabolite changes with M protein before and after treatment.** The changes of metabolites in MM patients before and after treatment were consistent with the levels of IgG or IgA type M protein, such as in aspartic acid, PE (18:1 / 18:2), lysoPE(16:0) and TG (18:1 / 18:1 / 22:5).

**Note:** (A) Changes of IgG type M protein before and after treatment; (B) Changes of IgA type M protein before and after treatment; (C) Aspartic acid;(D) PE(18:1/18:2);(E) LysoPE(16:0);(F) TG(18:1/18:1/22:5).

**FIGURE S12. Correlation analysis of BMPC and metabolites in MM patients.**

**Note:** BMPC, plasma cell in bone marrow; **(A)**, Aspartic acid（R^2^=0.2730，P=0.0151）; **(B)**, PC(19:0/22:2) （R^2^=0.0312，P=0.4559）; **(C)**, Hex2 cerd(18:2/24:0)（R^2^=0.0119，P=0.6382）; **(D)**, PC(16:0/16:0) （R^2^=0.2732，P=0.0181）; **(E)**, LysoPC(20:0) （R^2^=0.0615，P=0.2916）; **(F)**, LysoPE（18:1）（R^2^=0.0333，P=0.4413）; **(G)**, PC(O-22:0/22:4)（R^2^=0.4389，P=0.0015）; **(H)**, PC(16:0/20:5) （R^2^=0.1291，P=0.1198）; **(I)**, PC（18:0/24:1）（R^2^=0.0869，P=0.2071）.
