# Supplementary material for: Serum Abnormal Metabolites for Evaluating Therapeutic Response and Prognosis of Patients With Multiple Myeloma
Source: Front Oncol. 2022 Feb 28;12:808290. doi: 10.3389/fonc.2022.808290 (PMC8919723; doi:10.3389/fonc.2022.808290)

**TABLE S1.Independent prognostic factors analysis in 46 MM patients by multivariate Cox Regression**


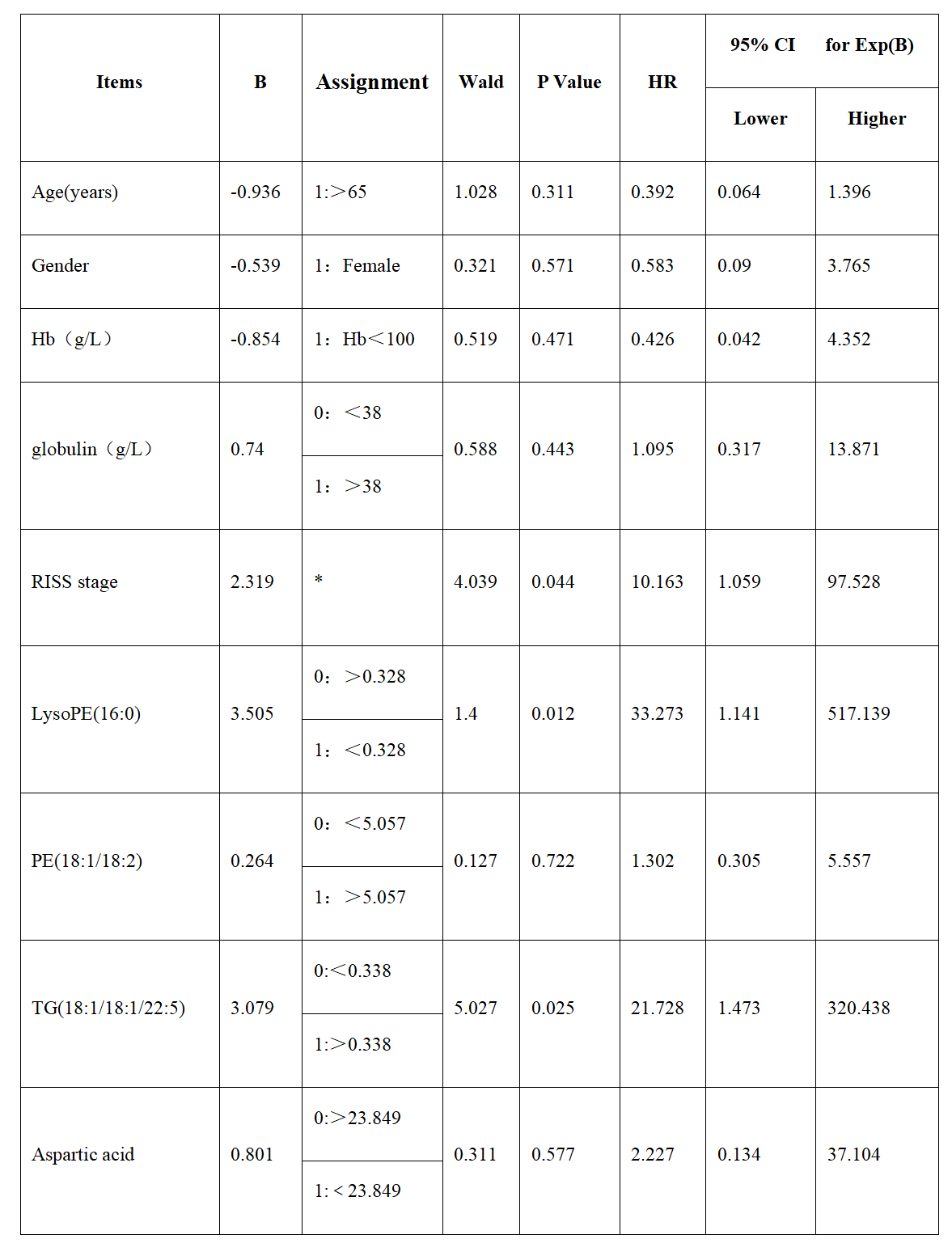

Supplement: Supplementary file 1 [file DataSheet_1.zip › Data Sheet 1/Table S1.docx]
