# Supplementary figures and images for: Serum Abnormal Metabolites for Evaluating Therapeutic Response and Prognosis of Patients With Multiple Myeloma
Source: Front Oncol. 2022 Feb 28;12:808290. doi: 10.3389/fonc.2022.808290 (PMC8919723; doi:10.3389/fonc.2022.808290)

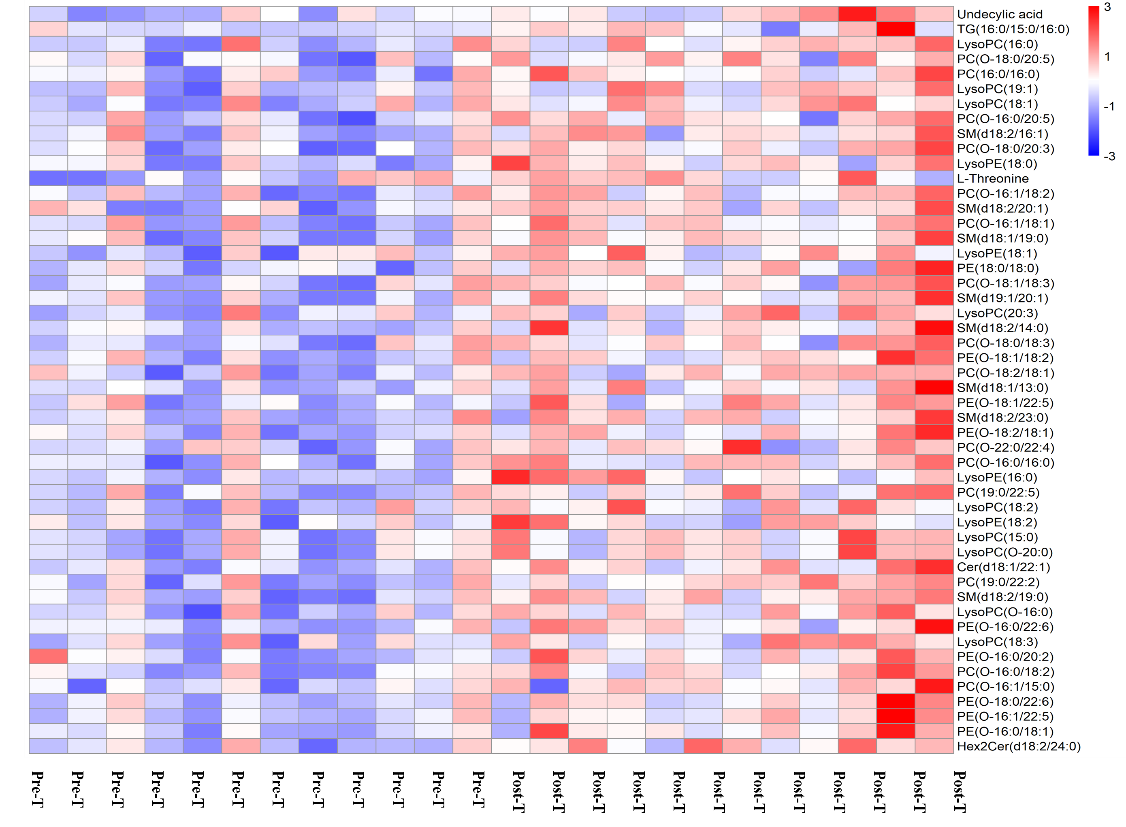

Supplement: Supplementary file 1 [file DataSheet_1.zip › Data Sheet 1/Figure S1.tif]

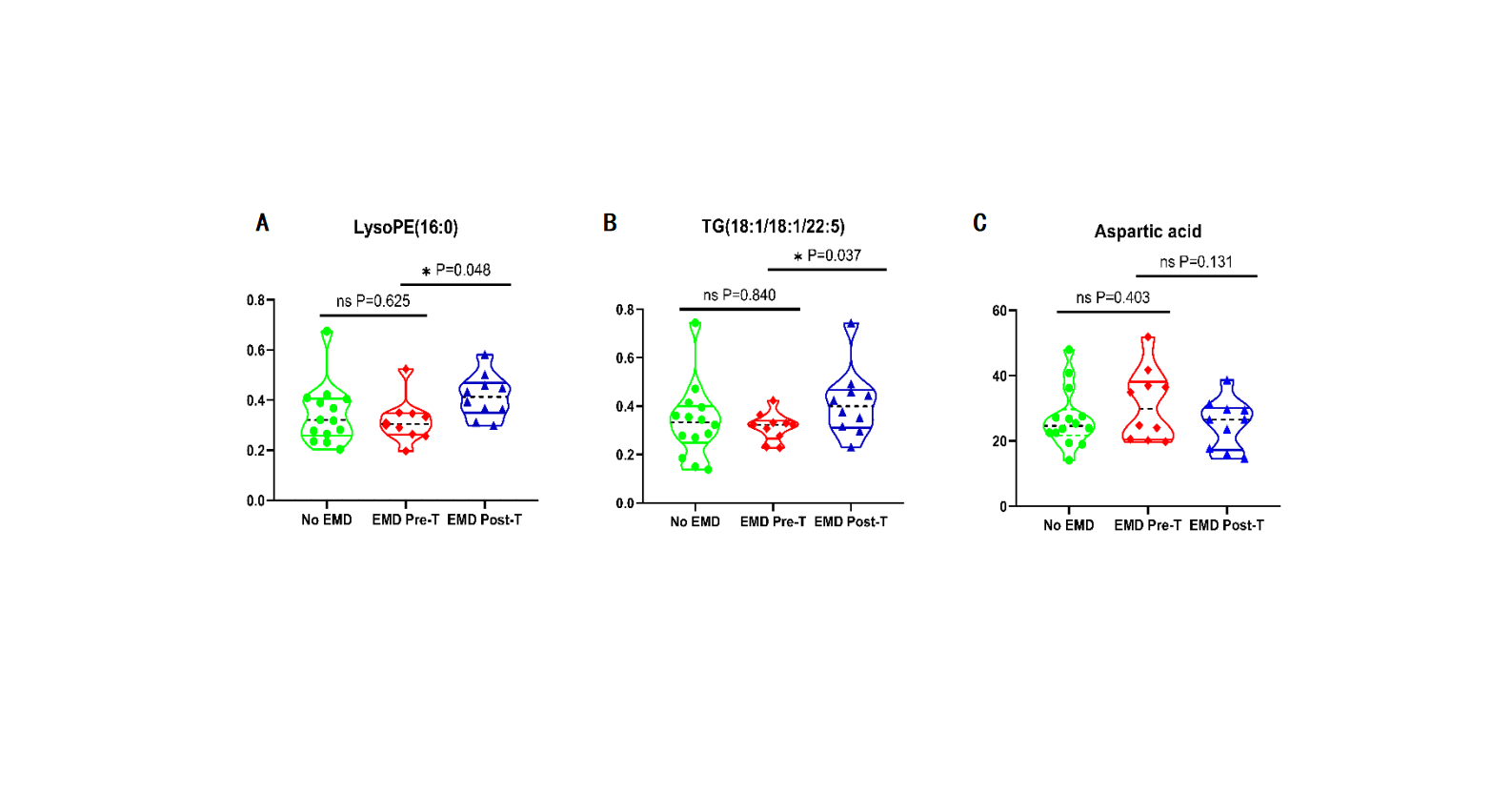

Supplement: Supplementary file 1 [file DataSheet_1.zip › Data Sheet 1/Figure S10.tif]

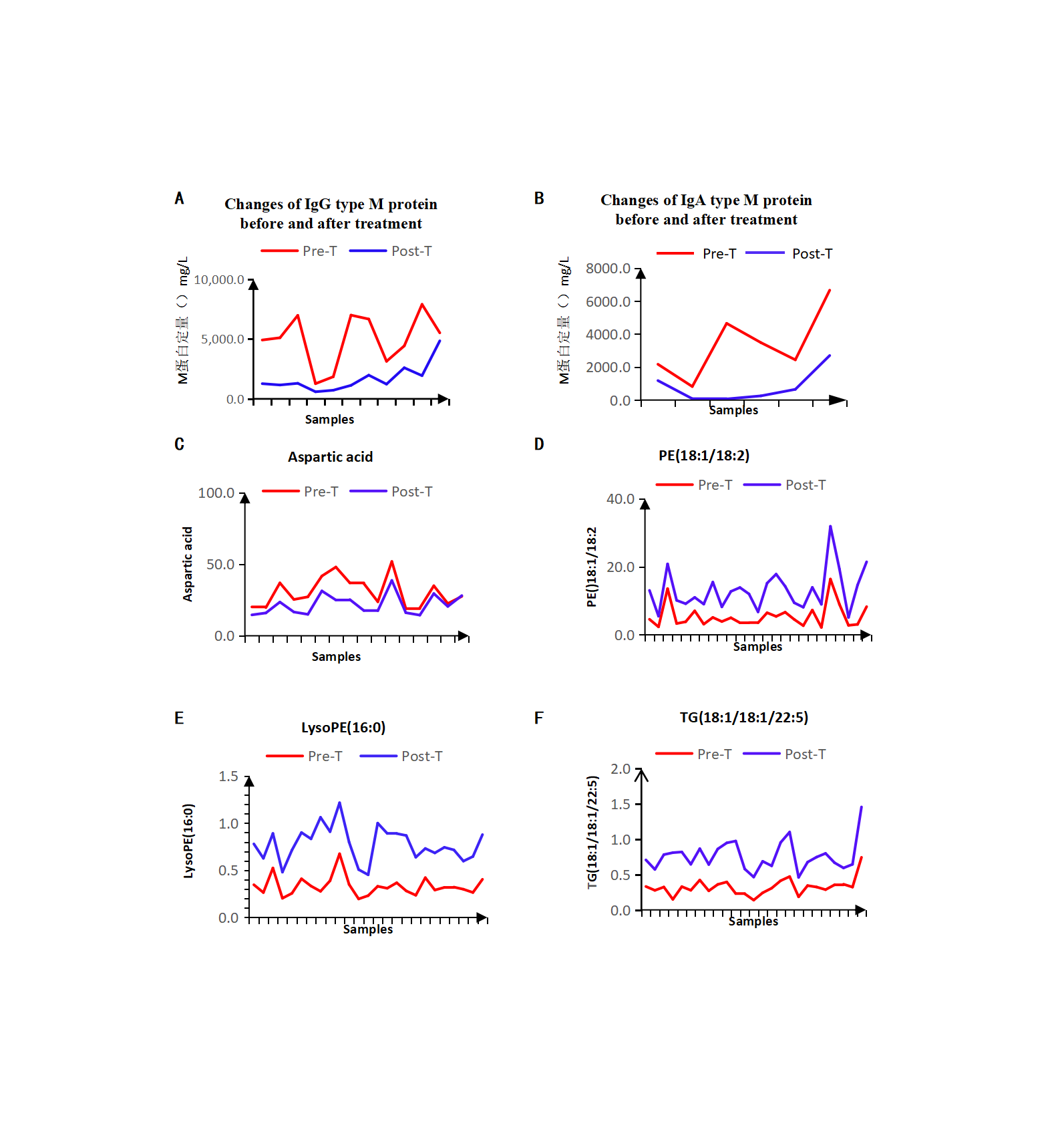

Supplement: Supplementary file 1 [file DataSheet_1.zip › Data Sheet 1/Figure S11.tif]

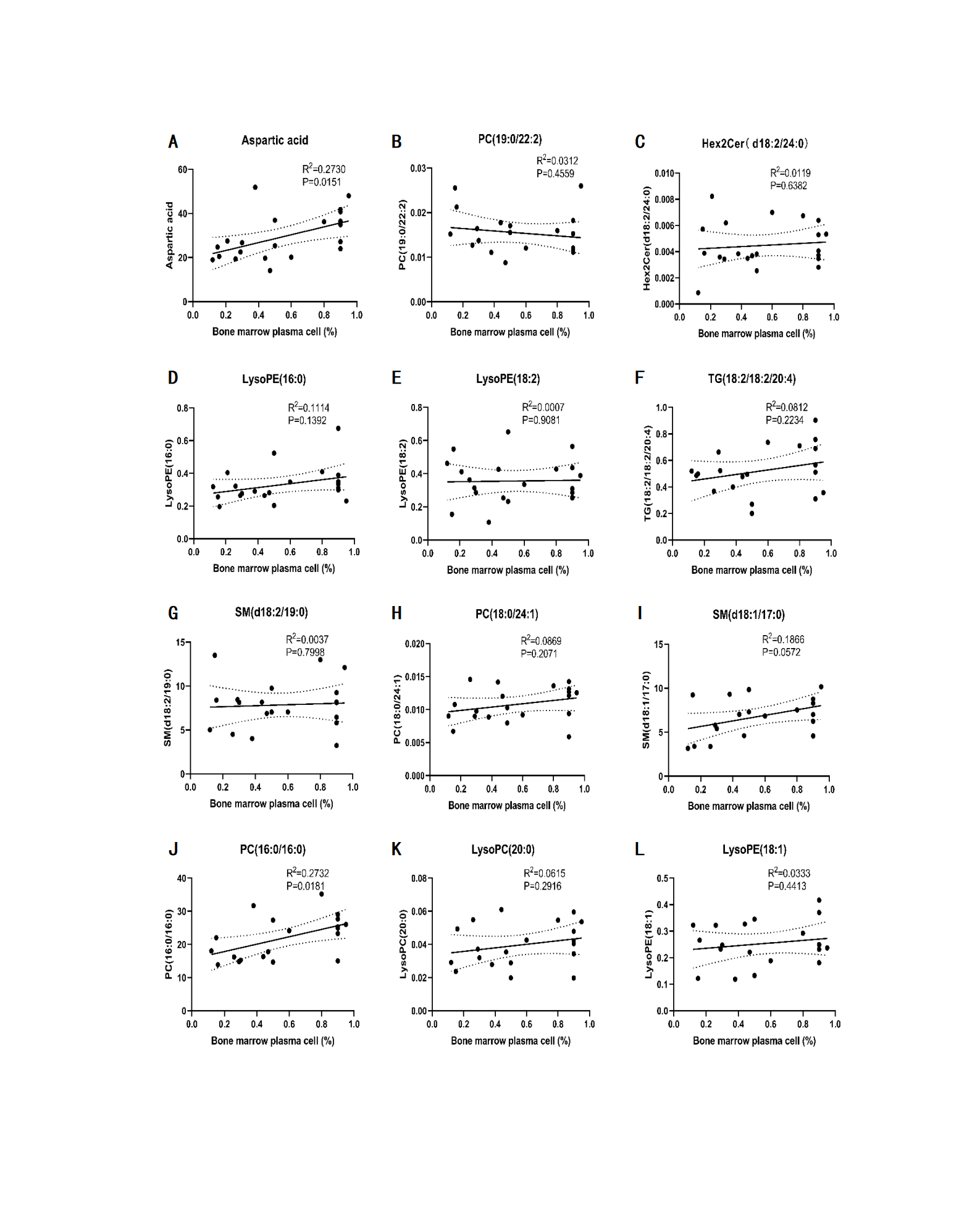

Supplement: Supplementary file 1 [file DataSheet_1.zip › Data Sheet 1/Figure S12.tif]

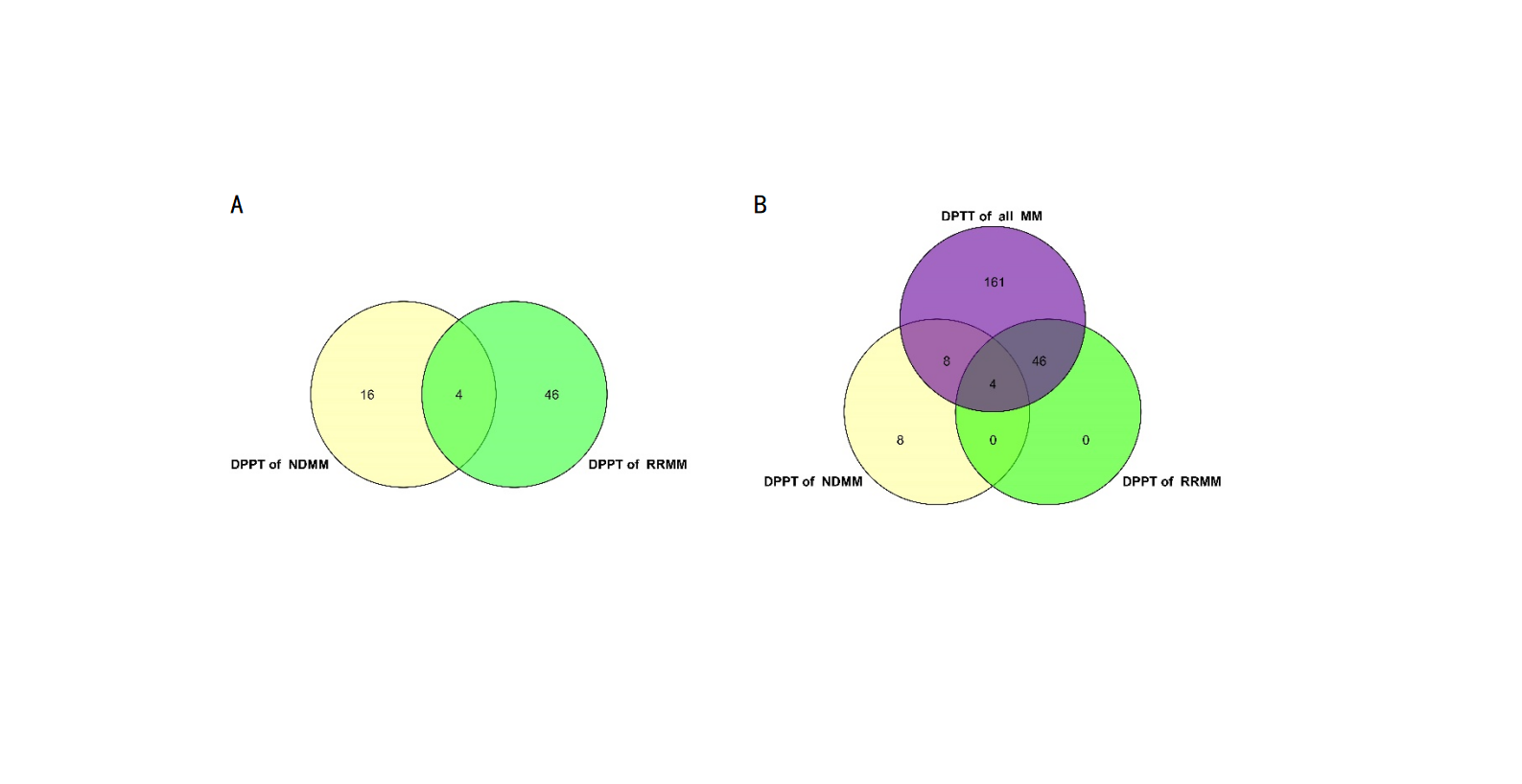

Supplement: Supplementary file 1 [file DataSheet_1.zip › Data Sheet 1/Figure S2.tif]

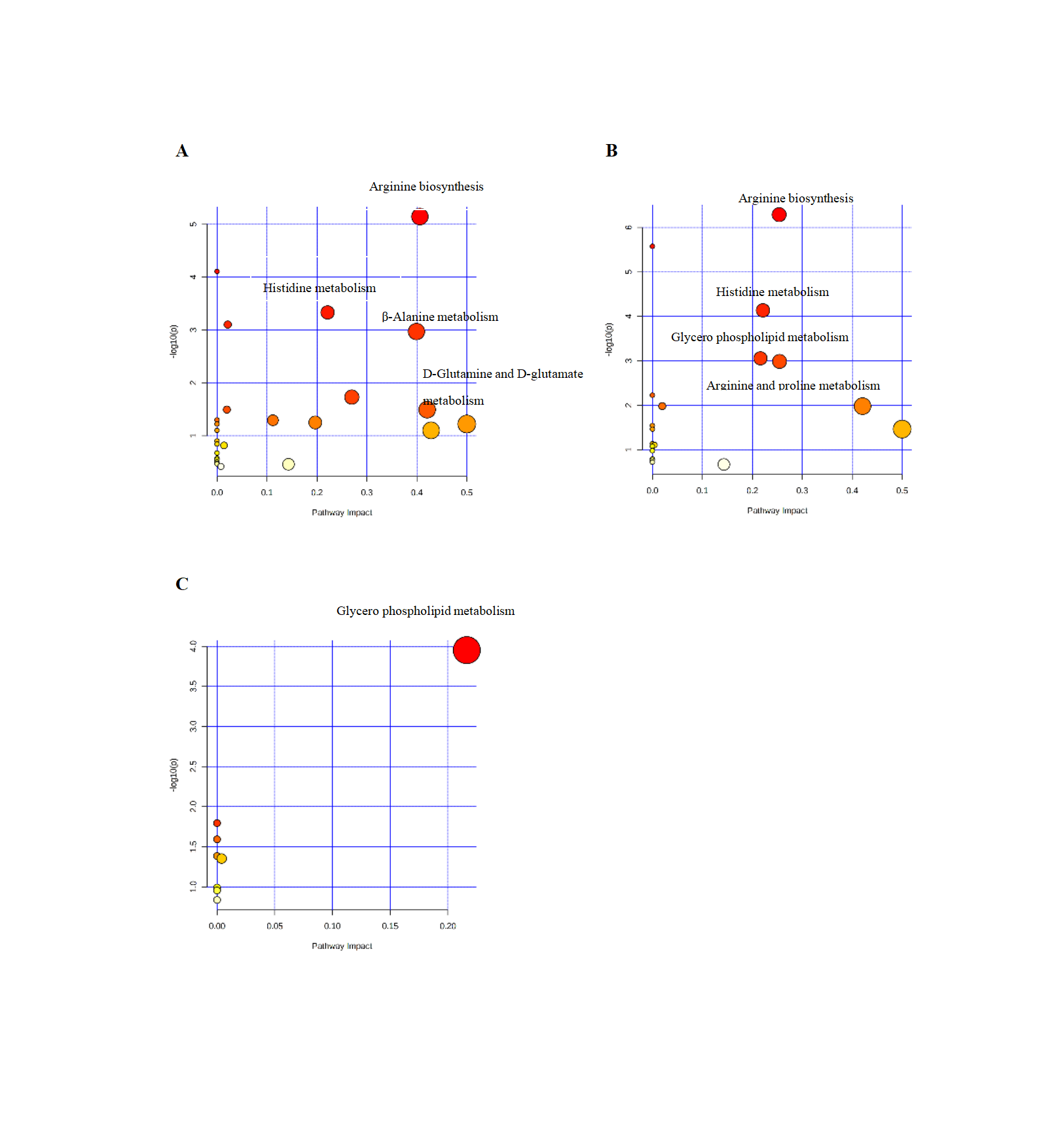

Supplement: Supplementary file 1 [file DataSheet_1.zip › Data Sheet 1/Figure S3.tif]

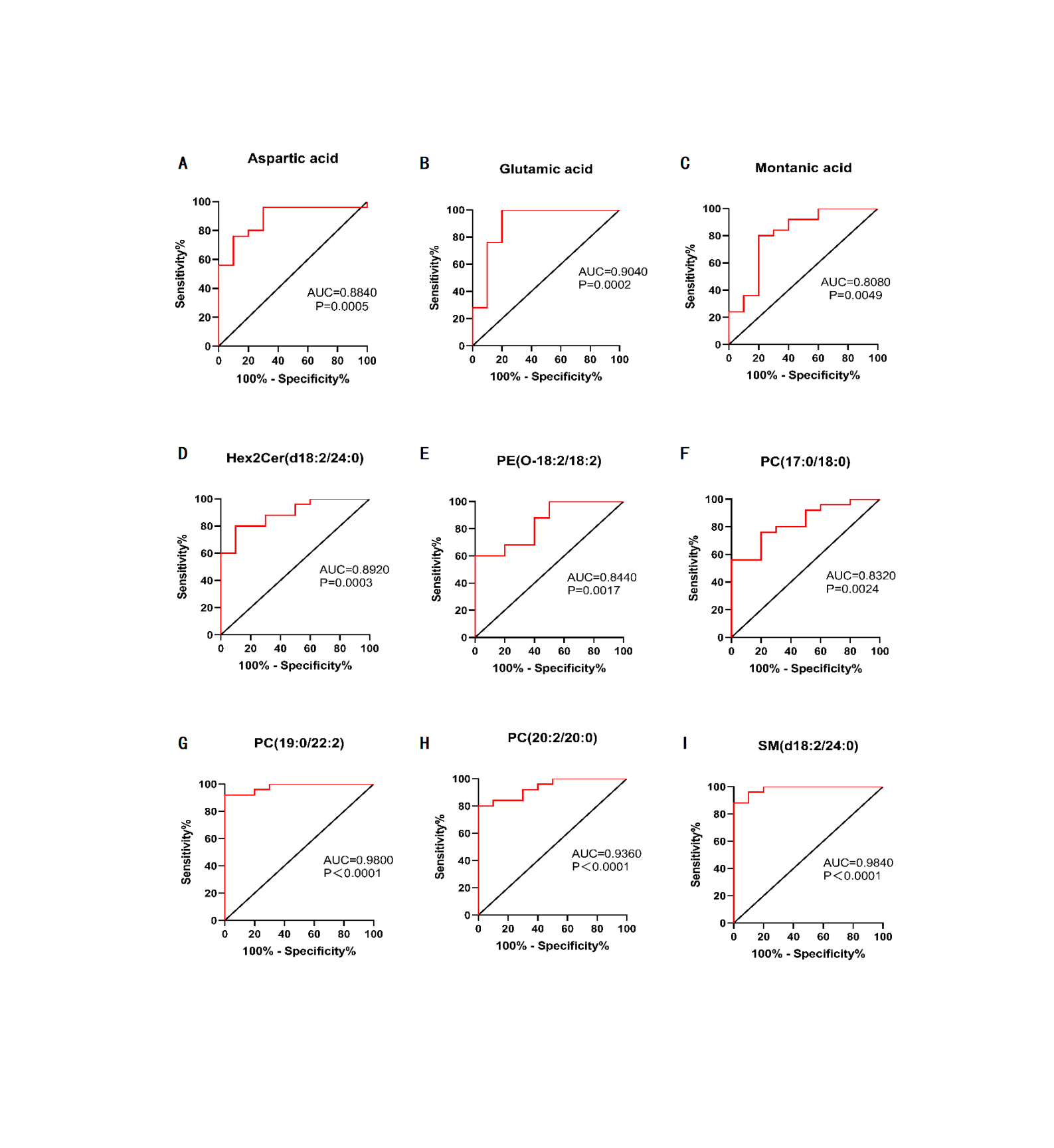

Supplement: Supplementary file 1 [file DataSheet_1.zip › Data Sheet 1/Figure S4.tif]

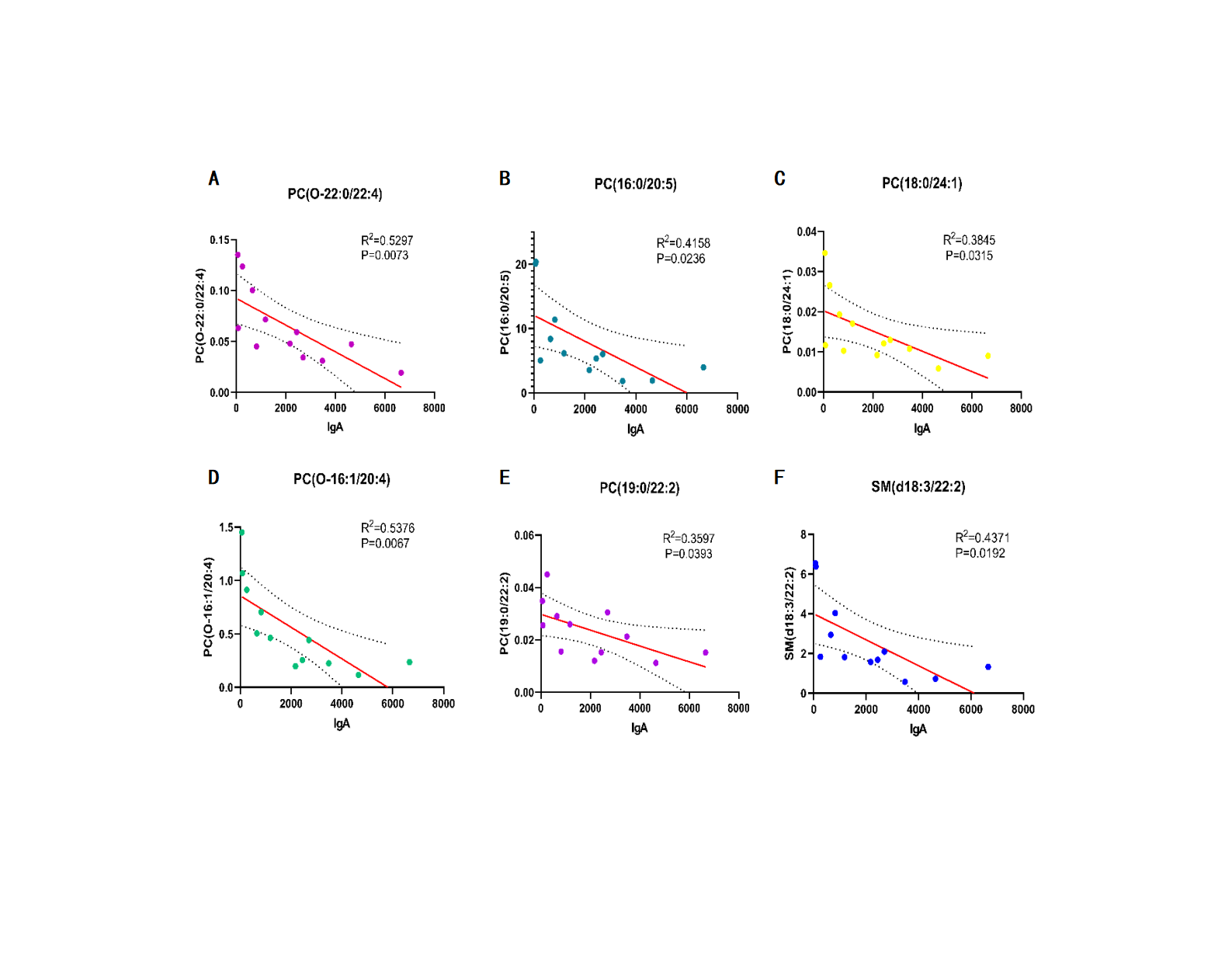

Supplement: Supplementary file 1 [file DataSheet_1.zip › Data Sheet 1/Figure S5.tif]

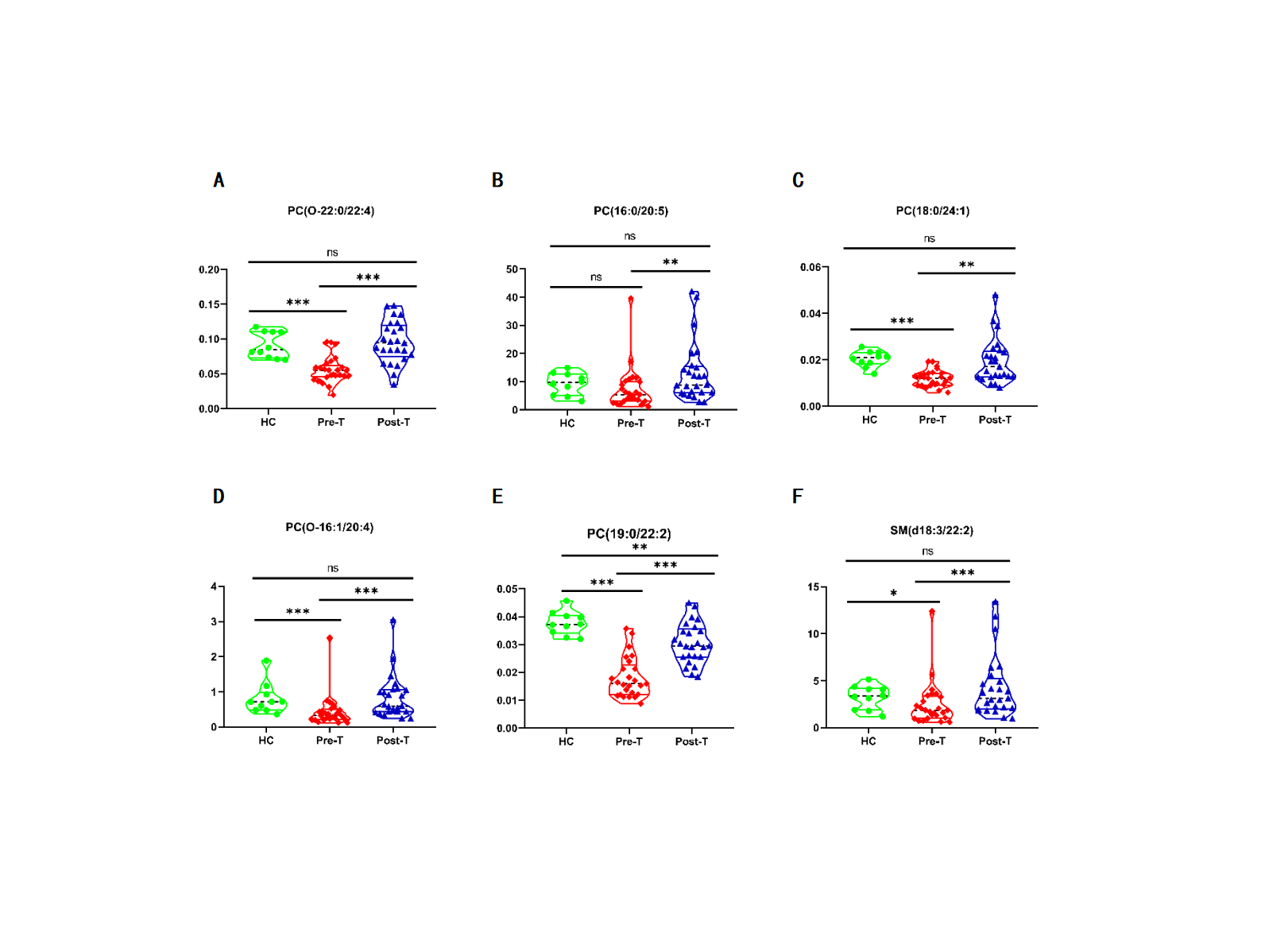

Supplement: Supplementary file 1 [file DataSheet_1.zip › Data Sheet 1/Figure S6.tiff]

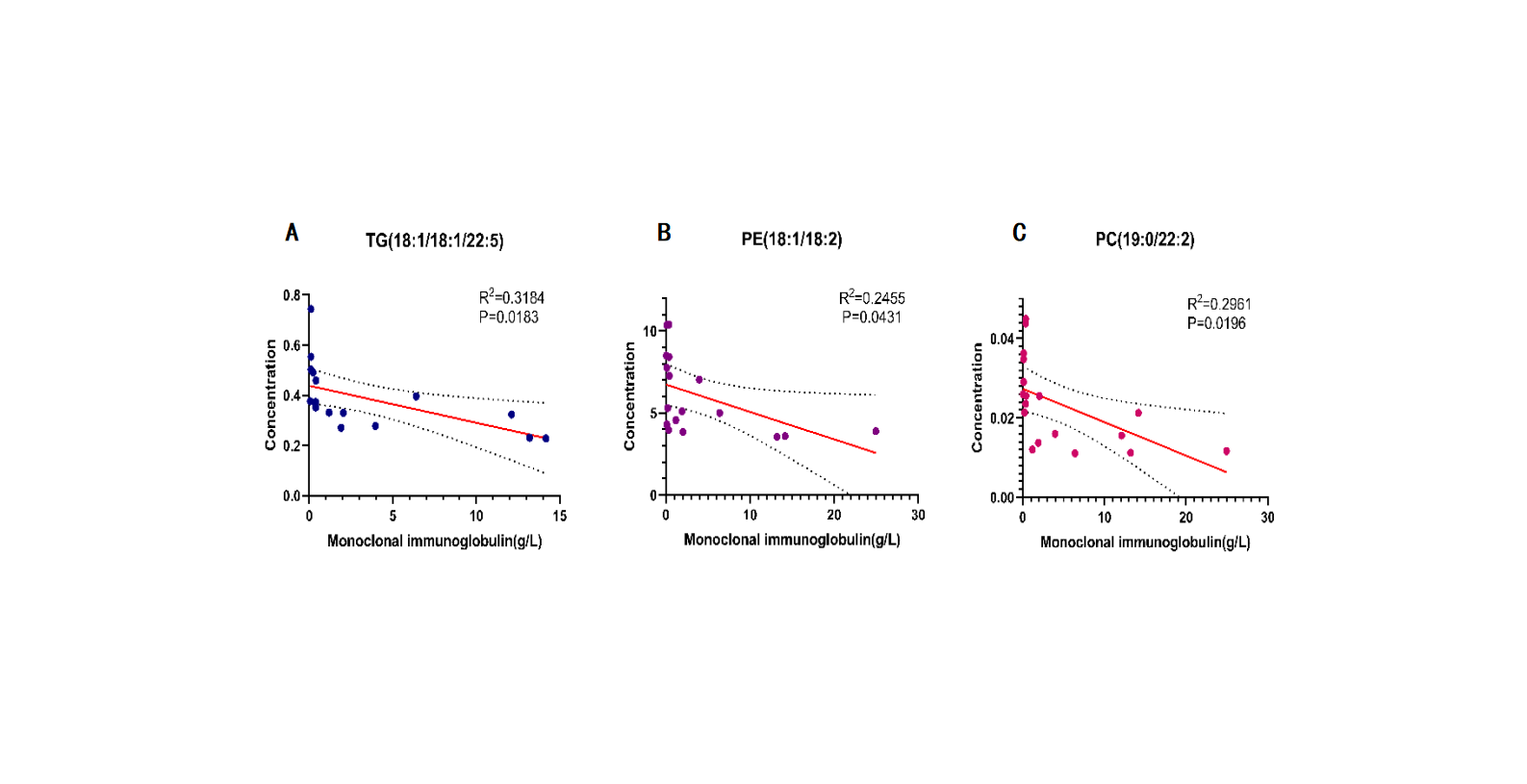

Supplement: Supplementary file 1 [file DataSheet_1.zip › Data Sheet 1/Figure S7.tiff]

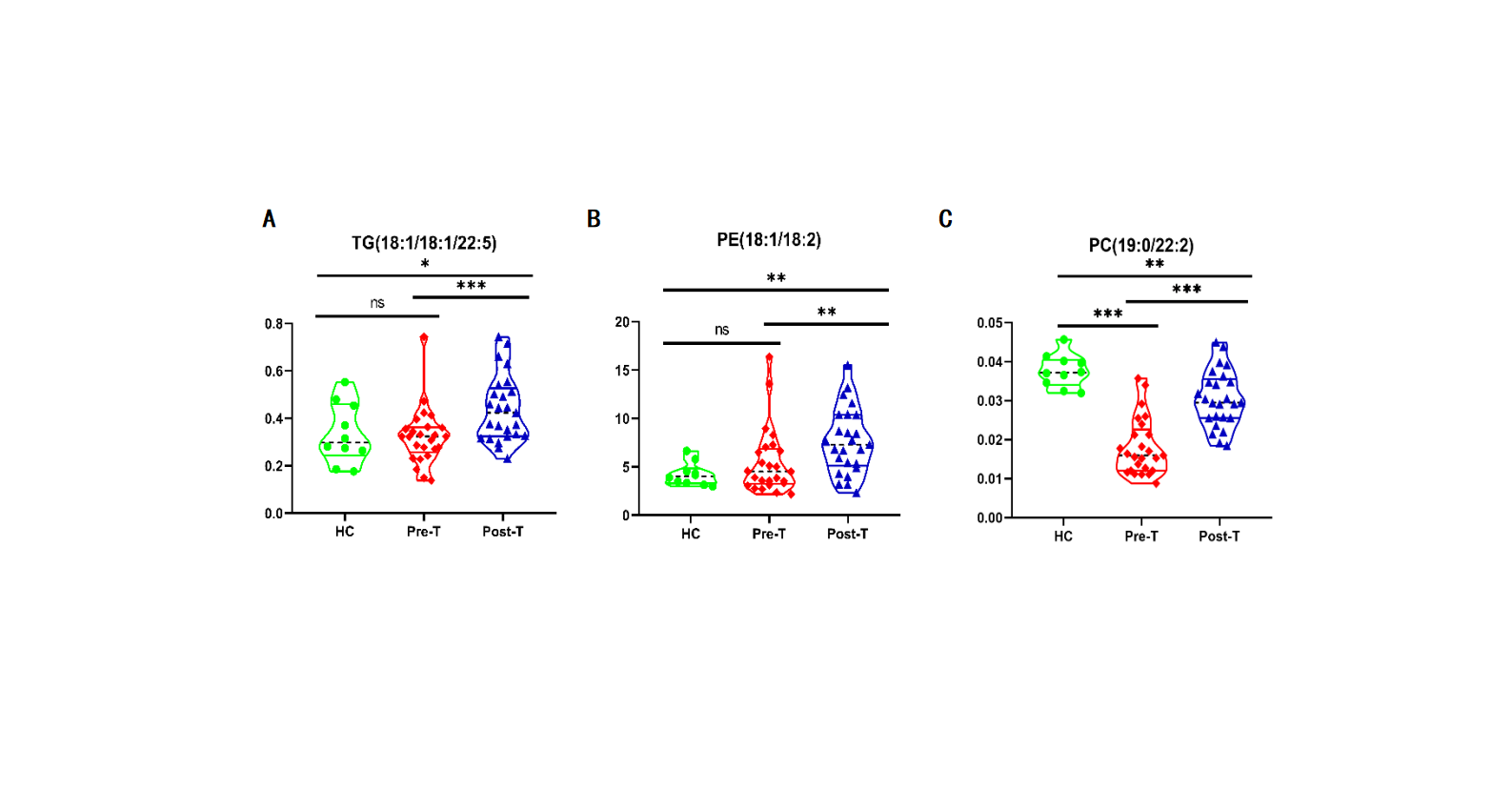

Supplement: Supplementary file 1 [file DataSheet_1.zip › Data Sheet 1/Figure S8.tiff]

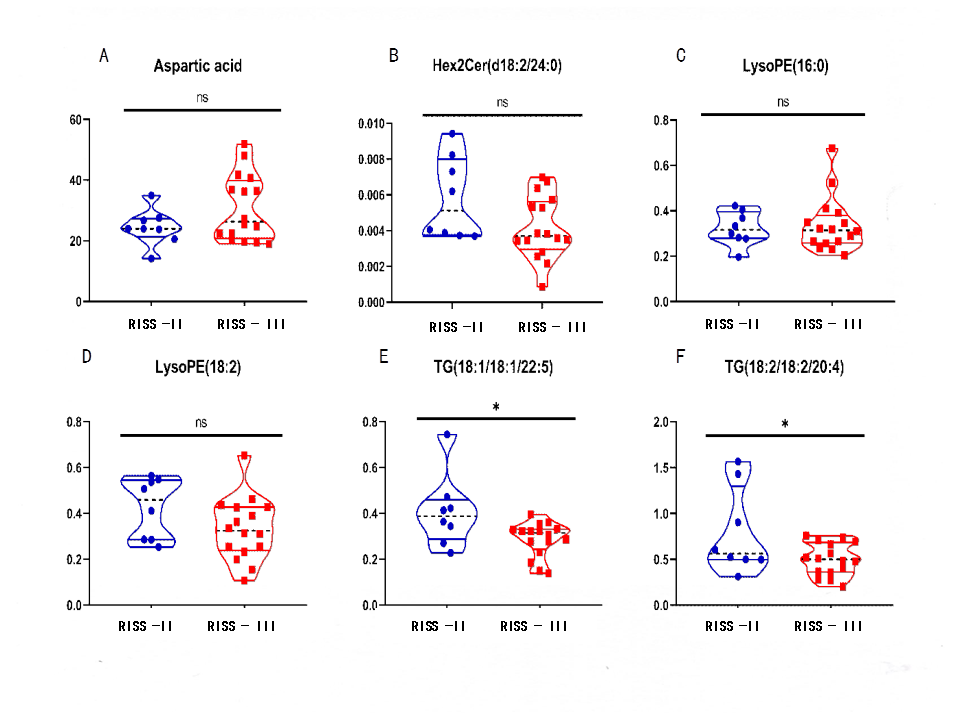

Supplement: Supplementary file 1 [file DataSheet_1.zip › Data Sheet 1/Figure S9.tif]
